# Three Decades of Change in Potentially Toxic Elements in Brown Algae in the Northeast Atlantic Ocean

**DOI:** 10.1021/acs.est.4c14013

**Published:** 2025-05-22

**Authors:** Carme Pacín, J. Ángel Fernández, Mercedes Conde-Amboage, Massimo Lazzari, Rita García-Seoane, Inés G. Viana, Zulema Varela, Carlos Real, Rubén Villares, Jesús R. Aboal

**Affiliations:** † CRETUS Centre, Department of Functional Biology, Ecology Unit, 16780 Universidade de Santiago de Compostela, Santiago de Compostela 15782, Spain; ‡ CIQUS Centre, Department of Physical Chemistry, Universidade de Santiago de Compostela, Santiago de Compostela 15782, Spain; § Department of Statistics, Mathematical Analysis and Optimization, Universidade de Santiago de Compostela, Santiago de Compostela 15782, Spain; ∥ 16534 Instituto Español de Oceanografía (IEO−CSIC), Centro Oceanográfico de A Coruña, A Coruña 15001, Spain; ⊥ Department of Earth Sciences, University of Hawaii at Ma̅noa, 1680 East-West Road, POST 719B, Honolulu, Hawaii 96822, United States; # Department of Functional Biology, Ecology Unit, Universidade de Santiago de Compostela, Escola Politécnica Superior de Enxeñaría, Lugo 27002, Spain

**Keywords:** Heavy metals, Seaweed, Biomonitoring, Marine pollution, Hazardous elements, Temporal trends

## Abstract

Marine pollution from potentially toxic elements (PTEs) threatens coastal ecosystems, making long-term assessments essential. This study analyzes trends in Al, Cr, Fe, Ni, Cu, Zn, As, Cd, and Hg using 446 samples of *Fucus ceranoides*, *F. spiralis*, and *F. vesiculosus* collected between 1990 and 2021 at 173 coastal sites in NW Spain. A consistent resampling approach revealed significant declines in most anthropogenic PTEs, including Cu (−84.7%), Cr (−84.6%), Hg (−49.6%), and Cd (−36.7%) over time. In contrast, arsenic increased by 36.1%, but the underlying causes remain unclear, with potential factors including changes in sediment inputs, bioavailability, or emerging sources such as groundwater discharges. Higher PTE levels were detected in inner estuarine areas, but no consistent latitudinal patterns emerged. Overall, the results suggest effective mitigation of coastal pollution, with reduced bioavailable PTEs entering the food web via *Fucus* spp. However, rising As levels and complex contamination dynamics underscore the need for continued monitoring. This study offers the most comprehensive standardized assessment of long-term PTE trends in brown algae to date, providing valuable insights for environmental policy and coastal management.

## Graphical abstract

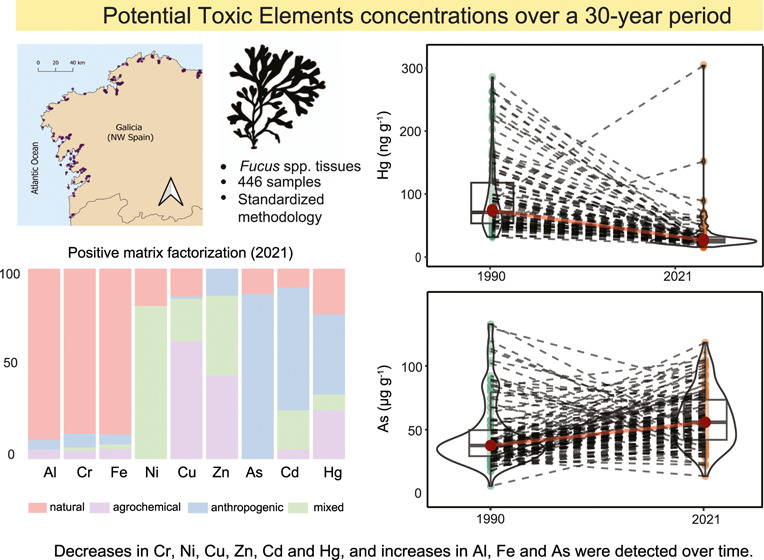

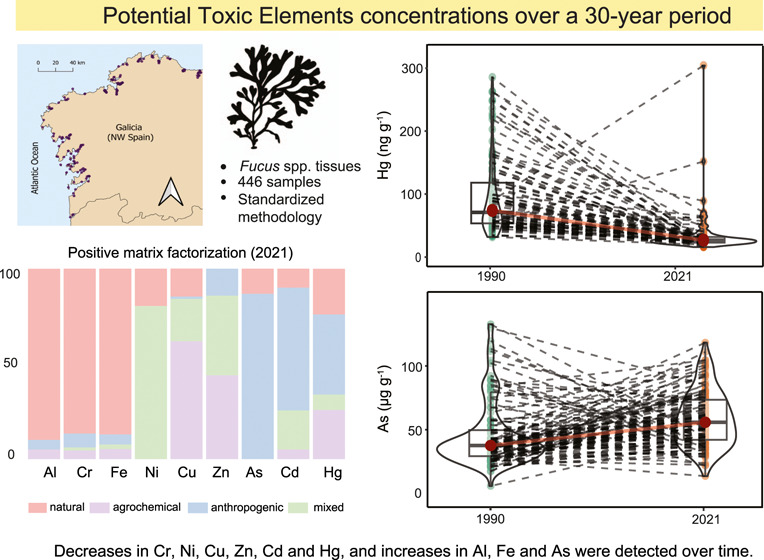

## Introduction

Pollution from Potentially Toxic Elements (PTEs) poses a significant threat to marine environments. PTEs are naturally occurring elements in the Earth’s crust that can be released into the marine environment through geological processes (e.g., volcanoes, erosion) and human activities (e.g., industry, mining, and fossil fuel combustion).^1^ The latter has intensified significantly since the Industrial Revolution, increasing discharges of these compounds.^2^ Some PTEs have metabolic importance and become toxic at high concentrations (e.g., Cu, Ni, and Zn), but others can be toxic even at trace levels (e.g., Hg and Cd).^3,4^

Brown algae (Phaeophyceae) accumulate PTEs at concentrations that often exceed those in seawater,^5,6^ leading to reduced reproduction and survival rates, and increased oxidative damage.^7–9^ This is critical given their role as ecosystem engineers in temperate coastal ecosystems, with *Fucus* species shaping intertidal habitats, acting as carbon sinks, and contributing to climate change mitigation.^10^ As primary producers, they transfer PTEs through the marine food web, potentially leading to biomagnification.^11–15^ The study of PTE concentrations in algae therefore holds intrinsic value due to their ecological significance.

Since the 1950s, PTE concentrations in brown algae have been used to assess marine pollution. Unlike water samples, which provide snapshots, and sediments, which record historical pollution, algal tissues reflect bioavailable PTEs and are therefore more indicative of ecological risk. Accordingly, comparisons of PTE concentrations in seawater and sediment with those in algae have often shown limited correlations.^16^ *Fucus* spp. have been especially valuable in the Northern Hemisphere because of their high accumulation capacity, wide distribution, and simple tissue structure.^17^

The use of brown algae as biomonitors has provided valuable insights into temporal trends in PTE pollution at global, regional, and local scales.^18–21^ However, these studies have often been compromised by the use of nonstandardized methodologies, the inclusion of different species, and inconsistent sampling seasons.^20^ Even studies applying standardized methodologies^22,23^ have often been limited by short observation periods and inconsistent or limited sampling sites.

Given the importance of identifying temporal trends for assessing the environmental impact of PTE pollution and the effectiveness of regulations, such as the Marine Strategy Framework Directive,^24^ comprehensive long-term studies using standardized methodologies are essential. For this purpose, environmental specimen banks, through the retrospective analysis of preserved samples, are a critical resource.^23,25^

This study aims to fill this gap in long-term pollution assessments of brown algae using standardized methodologies and a representative number of sampling sites by analyzing concentrations of Al, Cr, Fe, Ni, Cu, Zn, As, Cd, and Hg in the tissues of *Fucus ceranoides, F. spiralis*, and *F. vesiculosus* that were systematically collected from consistent sites in NW Spain over three decades (1990–2021). The samples, stored in the Galician Environmental Specimen Bank (Universidade de Santiago de Compostela), were analyzed (or reanalyzed) using consistent methodologies. The NW Spanish coast, a heavily navigated maritime route, has faced significant industrial development and pollutant discharge,^26,27^ but has also been subjected to European environmental policies aimed at reducing pollution.^28^ This combination of historical pressures and progressive management makes it an ideal study area, with findings potentially applicable to other coastal regions facing similar challenges. We hypothesized that a) regional PTE concentrations in *Fucus* spp. tissues will remain unchanged over time; b) PTE concentrations in *Fucus* spp. tissues collected from the same sites will remain unchanged over time; and c) PTE pollution sources, inferred from *Fucus* spp. PTE concentrations, will be stable over time.

## Material and methods

### 2.1 Study Area

Galicia region (NW Spain) features 1498 km of coastline dominated by rías, coastal inlets where the oceanic influence dominates, except in the inner estuarine zones (see Figure 1). The region has an oceanic climate with mild temperatures and consistent rainfall. Coastal industries include automotive, naval, energy, ceramics, metallurgy, and paper production.^29^ The region’s geology is predominantly granitic.^30,31^

**Figure 1:**
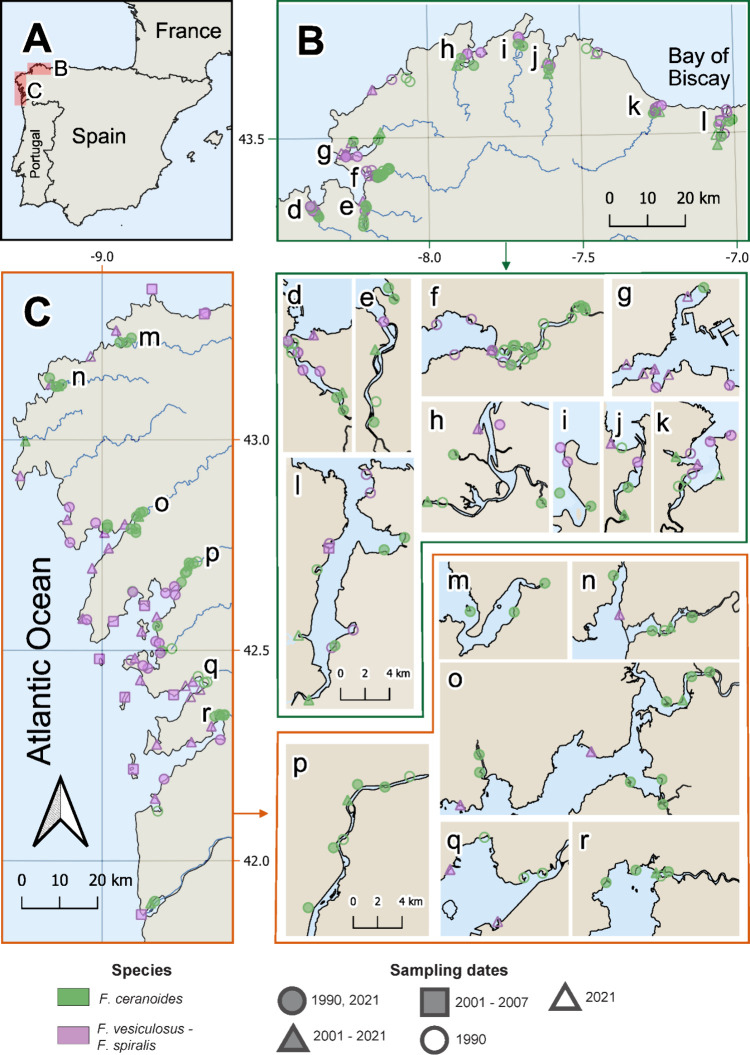
Map of the study area in NW Spain. Panels A–C display an overview of the region, with B and C showing the sampling sites. Panels d–l and m–r present detailed views of coastal inlets that cannot be distinguished in B and C, respectively. Colors represent the species sampled (*Fucus ceranoides* and *F. vesiculosus – F. spiralis*), while symbols indicate the sampling dates: circles for sites sampled in both 1990 and 2021, triangles for the 2001–2021 series (2001, 2003, 2005, 2007, 2021), rectangles for the 2001–2007 subset (2001, 2003, 2005, 2007), and unfilled circles and triangles for sites sampled in 1990 and 2021, respectively. To reduce clutter, incomplete samplings from 2001, 2003, 2005, and 2007 are grouped under the 2001–2007 symbol.

### 2.2 Sampling

A total of 446 samples of *Fucus ceranoides, F. spiralis*, and *F. vesiculosus* were collected from 173 sites in 1989–1990 (1990 from now on), 2001, 2003, 2005, 2007, and 2021 (Figure 1, Table S1). Most sites sampled in 1990 were resampled in 2021, and those sampled in 2001 were revisited in 2003, 2005, 2007, and 2021.

Due to uncertainties in distinguishing *F. spiralis* from *F. vesiculosus*, including potential hybridization and misidentification with *F. macroguiryi*,^32^ these taxa were treated as a single group, while *F. ceranoides* was categorized separately.

Sampling was conducted in July to minimize seasonal effects.^22^ At each site, a minimum of 30 thalli were collected at low tide in a zigzag pattern along a 50 m transect, rinsed on-site with seawater, combined into a composite sample, and transported to the laboratory in a cooler. Detailed sampling protocols were described elsewhere.^17^

### 2.3 Sample Processing

To minimize intrathallus variability in PTE concentrations,^22^ apical tissues corresponding to 3 dichotomous sections, which represent the recent growth period of the algae,^33^ were selected excluding receptacles and tissues with epiphytes. Samples were dried in a forced-air oven at 40 °C until constant weight, homogenized in a tangential mixer mill with zirconium oxide vessels (Retsch MM400), and stored in sealed glass vessels at room temperature, protected from light in the Galician Environmental Specimen Bank at Universidade de Santiago de Compostela until chemical analysis.

### 2.4 Chemical Analysis

Samples were dried again at 100 °C (Al, Cr, Fe, Ni, Cu, Zn, As, and Cd) or 40 °C (Hg). Samples from 1990, 2001, 2003, 2005, and 2007 were reanalyzed,^23^ while 2021 samples were analyzed for the first time. This approach allowed us to apply consistent and up-to-date methodology across the entire data set. Al, Cr, Fe, Ni, Cu, Zn, As, and Cd concentrations were determined by inductively coupled plasma mass spectrometry (ICP–MS, Agilent 7700x) at the Research Support Services Unit of the Universidade de Santiago de Compostela. Hg concentrations were measured in an elemental analyzer (Milestone DMA 80) at the Ecology unit in the same university.

Certified reference material (Bladderwrack-*Fucus vesiculosus*, ERM-CD200, Belgium), analytical blanks, and replicates were included every 30 samples for all PTEs, except for Hg, whose controls were included every 15. Recoveries ranged from 90% (for Cu) to 110% (for As), with Relative Percent Differences below 9% for all PTEs. Determinations were above the limit of quantification except for one sample for Cu and Al, and 19 samples for Cr (i.e., 4% of the total). Detailed analytical quality results are shown in Table S2.

### 2.5 Statistical Analysis and Visualization

Descriptive statistics and tests were conducted using R v4.1.1,^34^ including normality assessment (Shapiro-Wilk test), and variance homogeneity testing (Levene test from the ‘car’ package^35^). Data visualization was performed using the ‘ggplot2’ and ‘ggstatsplot’ packages.^36,37^ Species PTE content was compared with the Wilcoxon rank-sum test (*‘wilcox.test’* function), while Kruskal–Wallis (*‘kruskal.test’* function) and Dunn’s (‘FSA’ package^38^) tests compared temporal PTE trends across all *Fucus* spp. and samples (n = 446). Paired comparisons (1990 vs 2021) used Wilcoxon paired tests (*‘wilcox.test’* function), while Friedman (*‘friedman.test’* function) and Durbin-Conover (‘PMCMRplus’ package^39^) tests were applied to analyze repeated measures data from 2001, 2003, 2005, 2007, and 2021. For sites where two species were present in a given year, comparisons were made between them in that year and across years. All posthoc p-values were adjusted via Benjamini-Hochberg (BH, p.adjust function).

Linear Mixed Models (LMMs) were applied (‘lme4’ package^40^) to account for fixed (year, species) and random (ría) effects on the PTE concentrations, and to properly model the potential correlations arising from the use of repeated measures. Data were grouped by ría due to the limited number of observations per site, typically from 1 to 3. Best-fitting models were selected based on Akaike Information Criterion (AIC) and Bayesian Information Criterion (BIC) values. Detailed information on the LMMs can be found in the Supporting Information.

Spearman correlation analyses (*‘cor.test’* function from ‘Hmisc’ package,^41^ and ‘polycor’,^42^ and ‘ggcorrplot’^43^ packages for visualization) on PTE data, with p-values adjusted using the BH method, and Principal Component Analysis (PCA) on log-transformed PTE values (‘FactoMineR’ package^44^), were conducted^44^ to assess relationships between PTEs. Additionally, because three time periods (i.e., 1990, 2001–2007, and 2021) were found to have significantly different values for PTE concentrations (see section 3.2), positive matrix factorization models (PMF) were performed separately for each period to estimate PTE sources and contributions (PMF5 software^45^). Twenty base models were run for each period, and the model with the lowest q-robust value was selected. Factor numbers were determined through Bootstrap.

Spatial variation of PTE concentrations was mapped with QGIS 3.36.3.^46^ Percentage change over time was calculated as the difference between the oldest and latest available concentrations at each site. In addition, overall median percentage changes were calculated, along with median concentrations at each site for each element. Bioconcentration factors (BCFs) were calculated to compare PTE concentrations in seawater and algae samples. Since seawater concentration data were only available for Al, Cd, Cr, Cu, Fe, Ni, and Zn in 2023,^16^ BCFs for these elements were derived by pairing the 2023 seawater data with the 2021 algae data, as follows:

1 $$\mathrm{BCF}=\frac{median{\left[PTE\right]}_{algae}}{median{\left[PTE\right]}_{seawater}}$$

Sediment contributions to PTE concentrations in algae were estimated using Al as a geological tracer,^47^ with PTE and Al ratios calculated from published sediment data,^23,48^ and corresponding algae measurements. Due to the unavailability of sediment data for 2021, this analysis was limited to the years 1990, 2001, 2003, 2005, and 2007. Sediment contributions were determined for each sampling site and year^23,48^ using the following equation:

2 $$\mathrm{sediment contribution}=\frac{\frac{{\left[PTE\right]}_{se\dim ent}}{{\left[PTE\right]}_{algae}}}{\frac{{\left[Al\right]}_{se\dim ent}}{{\left[Al\right]}_{algae}}}$$

The median sediment contribution was calculated for each available PTE and year.

## 3 Results

### 3.1 Data Contextualization

PTE concentrations were non-normally distributed and skewed to the right. Median PTE concentrations ranged from 0.023 to 290 μg g^–1^, following the sequence: Hg < Cd < Cr < Ni < Cu < As < Zn < Al < Fe (Table S3). Detailed PTE concentrations by species, site, and year are presented in Table S4.

PTE concentrations varied significantly between species, with the exception of Cd. *Fucus ceranoides* exhibited higher concentrations of all PTEs (*p* < 0.01), with the exception of As, which was higher in *F. vesiculosus* – *F. spiralis* (*p* < 0.05) (Table S3). Spatially, PTE concentrations were usually higher in the inner part of the rías (Figures S4–S9), although this pattern was less pronounced for Hg and absent for Cd and As (Figures S1–S3). Only Ni concentrations exhibited latitudinal differences, with higher values in the northern rías (Figure S7B). Nearby rías did not show similar PTE concentrations. Some rías showed elevated levels of specific elements (e.g., rías ‘e’, ‘k’, and ‘o’ for Hg in Figure S1, or ‘i’, ‘j’, and ‘o’ for Cd in Figure S2), while others exhibited high within-ría variability, especially for As (Figure S3). Accordingly, the coefficient of dispersion (COD; i.e., median absolute deviation divided by the median) was elevated for all the PTEs (Table S3). Additionally, high concentrations in certain rías were not consistent across all elements, indicating substantial interelement variability (e.g., ría ‘p’ for Hg and Zn in Figures S1 and S9).

BCFs indicated that *Fucus* spp. concentrations were much higher than those in seawater, with the exception of Zn. Sediment contributions varied among PTEs and increased significantly since 1990 (Table S5).

### 3.2 Long-Term Trends

Median PTE concentrations exhibited decreasing Cr, Ni, Cu, Zn, Cd, and Hg, and increasing Al, Fe, and As levels over the study period (Table S3). These trends were supported by Kruskal–Wallis tests with Dunn’s posthoc comparisons, which revealed significant changes for all PTEs between 1990 and 2021. Concentrations remained largely stable during 2001–2007, with minimal intraperiod variation. Comparisons between 1990 and 2007 and 2001–2021 periods showed fewer but still notable significant differences (Table S3). Complementary analyses using Friedman tests with Durbin-Conover posthoc comparisons (2001–2021), and Wilcoxon paired tests (1990 and 2021), restricted to sites with repeated sampling across years, were very similar, confirming these patterns (Figure 2, Figures S10–S11). Friedman tests were insignificant for Al (p = 0.42) and near significant for Cd (p = 0.06), prompting exploratory Dunn’s tests for Cd.

**Figure 2:**
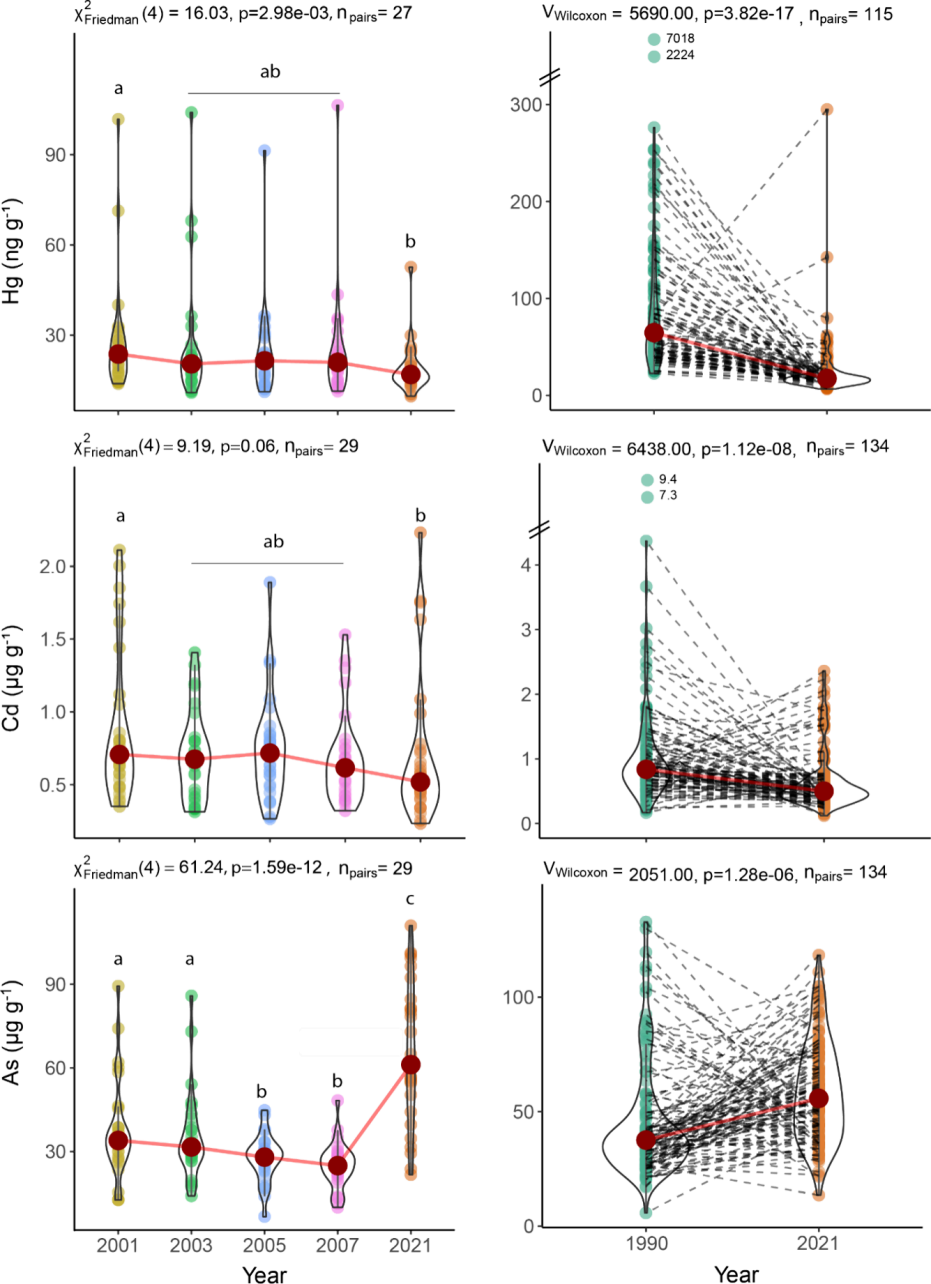
Temporal trends of Hg (ng g^–1^), Cd (μg g^–1^), and As (μg g^–1^) concentrations in *Fucus* spp. Left panel: Repeated measures (2001–2021) analyzed by Friedman test with Durbin–Conover posthoc comparisons. Right panel: Paired 1990 vs 2021 comparisons (Wilcoxon test). Distinct lowercase letters indicate significant differences between years (no shared letters = significant). Note: For Cd, the Kruskal–Wallis test was marginal nonsignificant (*p* = 0.06); however, Dunn’s posthoc test was conducted for exploratory purposes.

Spatial-temporal interactions were further assessed using LMMs, which confirmed significant decreases in all PTEs except Al, Fe, and As, which increased. Best-fit models (based on AIC/BIC) varied by element: random slopes (year|ría) were included for Hg, Cr, Ni, Cu, Zn, and As; Al, Fe, and Cd models used ría as a random intercept (1|ría), and all of them except Cd included species as a fixed effect. Detailed LMM outputs are shown in Table S6. Median percentage changes, derived from site-level changes from the first to last survey, showed decreases in Cr (−84.7%), Ni (−72.4%), Cu (−84.7%), Zn (−24.4%), Cd (−36.7%), and Hg (−49.6%), while Al (+367.9%), Fe (+105.2%), and As (+36.1%) increased (Figure 3 and Figures S12–S19). No consistent latitudinal or inner-outer rías patterns emerged, though localized substantial increases were detected in certain rías (e.g., Cd for ría ‘m’ and Cr for ría ‘h’, Figures S12 and S15), and specific sites from 1990 to 2021 (e.g., Cd, As, Ni, and Cu in ría ‘h’; Zn, Cd, Cr, As in ría ‘m’; and Hg in rías ‘h’ and ‘d’).

**Figure 3:**
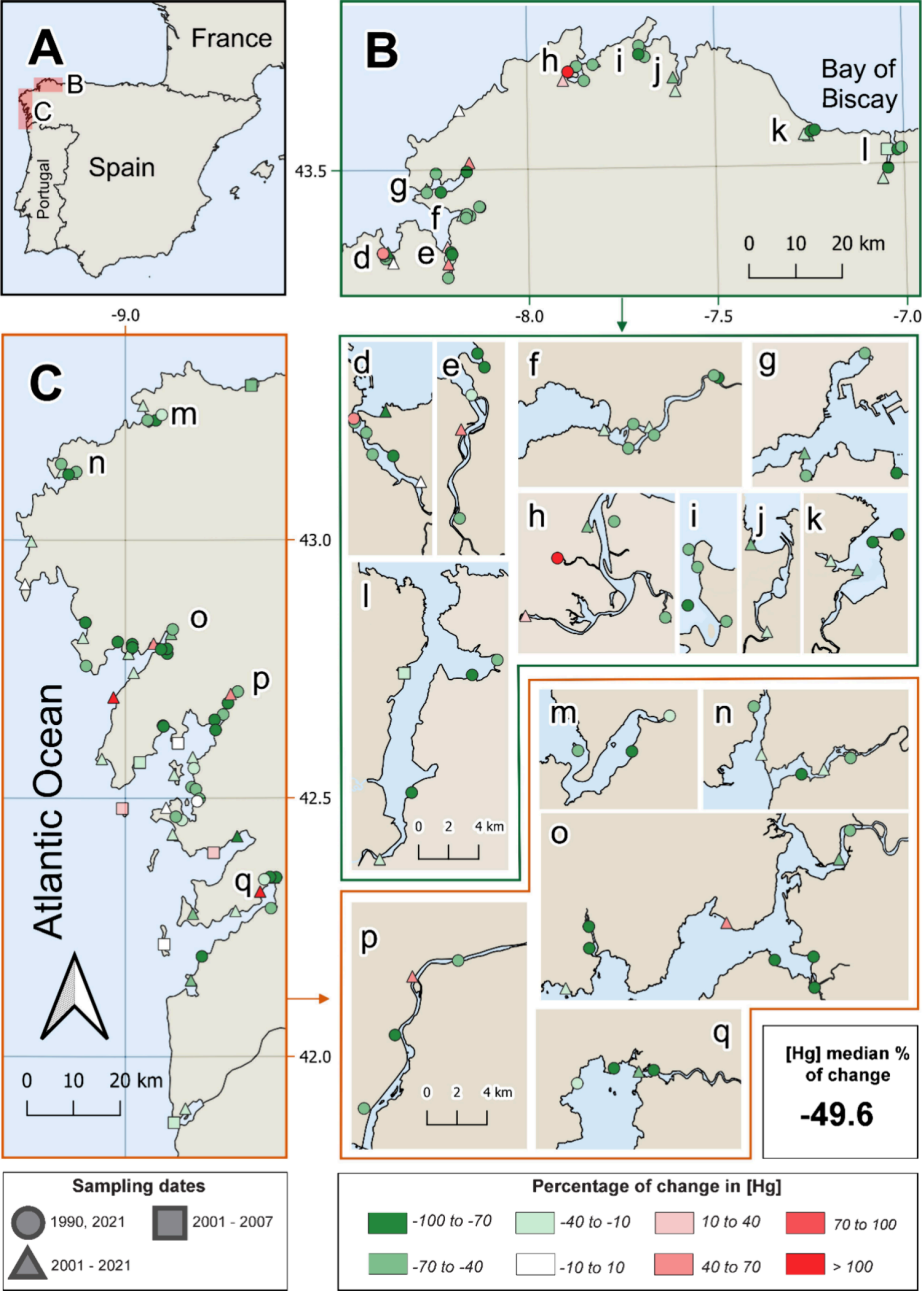
Map of percentage changes in Hg concentrations over time. Panels A–C provide a regional overview, with B and C showing the differences between the final and initial Hg concentrations (in %) at each sampling station. Panels d–l and m–q offer detailed views of sampling sites that are densely clustered and hard to distinguish in B and C, respectively. Different colors represent the percentage changes in Hg concentrations, while distinct symbols indicate the sampling dates: 1990 and 2021, 2001–2021, and 2001–2007. The total median percentage change, calculated as the median of the percentage changes across all sampling sites, is displayed below.

### 3.3 Identification of Potential Sources

Correlation analysis revealed strong positive relationships between Al–Fe, Cr–Ni, Cu–Cr, and Cu–Ni (*r* > 0.55), while As showed a moderate positive correlation with Al (r = 0.23), but negative correlations with Cu, Cr, and Ni (r ≈ −0.25) (Figure S20A). PCA supported these patterns, with PC1 dominated by Cr, Ni, Cu, Zn, and Hg and PC2 by Fe and Al, explaining 58.4% of the variance. As and Cd clustered separately (Figure S20B). PMF models identified four consistent factors across 1990, 2001–2007, and 2021, with shifts in element allocation, especially in 2021. In 1990 and 2001–2007, Factor 1 represented Al and Fe, Factor 2 Zn, As, Cd, and Hg, Factor 3 Cr and Ni (though Ni showed mixed contributions in 2001–2007), and Factor 4 Cu. In 2021, Factor 1 included Fe, Al, and Cr, Ni shifted to Factor 3, As, Cd, and Hg remained in Factor 3, Zn was distributed between Factors 3 and 4, and Cu remained primarily associated with Factor 4, with contributions from Factors 1 and 3 (Figure 4, Table S7).

**Figure 4:**
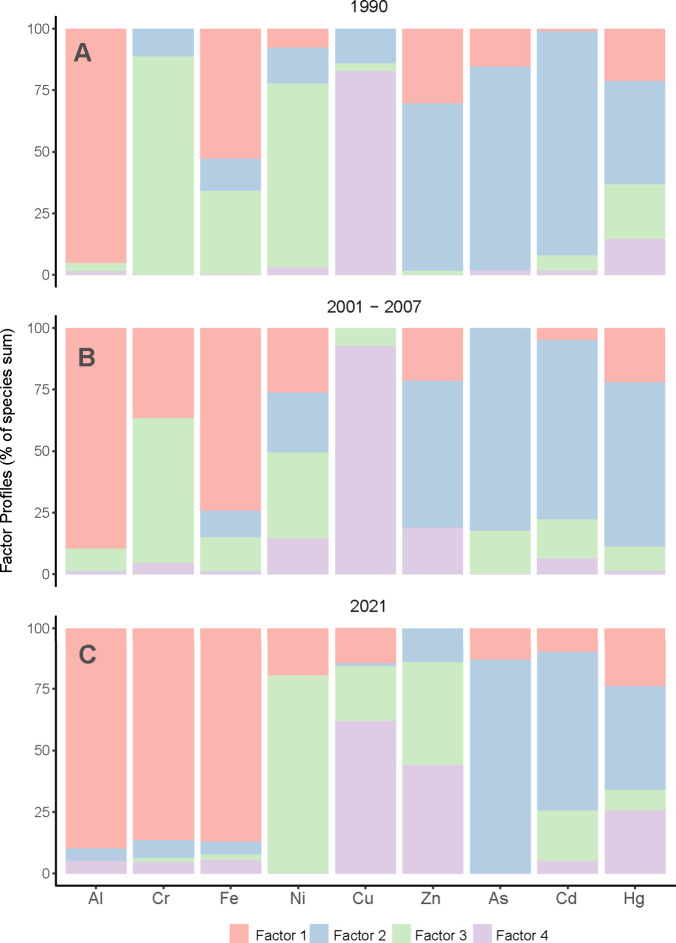
PMF results. Percentage contribution of each element to the factors identified using the Positive Matrix Factorization method. Panel A shows the results for 1990, panel B for 2001–2007, and panel C for 2021.

## 4 Discussion

### 4.1 PTE Concentrations and Spatial Patterns

Median PTE concentrations in *Fucus* spp. were largely consistent with values from a global meta-analysis,^20^ suggesting potential physiological regulation of metal composition. However, the observed higher levels of Cu (+66%) and As (+21%), and lower levels of Cd (−28%) compared to the global medians suggest potential regional differences in bioavailability and anthropogenic influences. Overall, these findings suggest moderate pollution levels in the area, with Cd and Hg medians within EU limits for seaweed consumption.^49,50^ *Fucus* spp. showed significantly higher PTE concentrations than regional seawater (except Zn), confirming their role as bioconcentrators of metals.^51^ Sediment contributions varied among PTEs and were higher in 2001–2007 than in 1990 (Table S5).

Interspecific comparisons revealed higher PTE concentrations in *F. ceranoides*, except for Cd and As (Table S3, Figures S1–S9). This pattern may reflect habitat-related factors rather than intrinsic differences in accumulation capacity.^47,48^ The observed spatial segregation (Figure 1), with *F.* ceranoides inhabiting sediment-rich estuarine environments and *F. vesiculosus – F. spiralis* occupying rocky marine substrates,^52,53^ likely contributes to greater sediment-derived inputs (as evidenced by elevated Al and Fe concentrations) and increased exposure to anthropogenic discharges typical of estuarine zones. Additionally, lower salinity and pH in these areas may enhance PTE bioavailability.^54,55^ In contrast, higher As levels in *F. vesiculosus – F. spiralis* likely reflect the naturally higher As concentrations in marine environments.^56,57^ Estuarine areas may exhibit lower As due to freshwater dilution and sedimentation.^58^

No clear spatial trend in PTE concentrations was found, with high variability among rías reflecting localized natural and anthropogenic influences (COD in Table S3; Figures S1–S9). Within-ría variability was marked, with no single ría consistently exhibiting high levels across all PTEs or sites. However, despite this variability, LMMs (Table S6), with ría included as a significant random effect, confirmed that each ría has a distinct PTE signature. For Al and Fe, random slopes for Year were not supported, likely because their concentrations, of lithogenic origin, are mainly driven by relatively stable sediment inputs, showing less interannual variability within rías. In the case of Cd, species was not included as a random slope, consistent with the lack of significant differences among species. This suggests a uniform accumulation pattern, with no additional species driven variation complicating spatial or temporal trends. Such homogeneity stabilizes Cd’s signal across *rías* and years, obviating the need for random slopes in the model.

### 4.2 Long-Term Trends and Source Apportionment

Temporal trends revealed significant decreases in Cr, Ni, Cu, Zn, Cd, and Hg with reductions ranging from −84.7% for Cu to −24.4% for Zn, alongside notable increases in Al (367.9%), Fe (105.2%), and As (36.1%). The most pronounced shifts occurred between 1990 and 2021, while concentrations remained relatively stable between1990–2001, 2001–2007, and 2001–2021 (Table S3, Figure 2, Figures S10 and S11). This underscores the critical role of long-term monitoring in detecting gradual trends that short-term studies may overlook. These trends are consistent with global declines in PTE concentrations in brown algae,^20^ as well as previous reports from the same study area on *Fucus* spp.,^22,23^ and other regional observations in these organisms.^18,21,59^ Despite the absence of latitudinal or inner-outer ría patterns, localized PTE increases emerged between 1990 and 2021, with Hg increasing in rías ‘h’ and ‘d’, Cd in ría ’m’, and Cr in ría ’h’ (Figure 3 and Figures S12 and S15). Specific sites showed multielement spikes (Cu, Ni, Zn, and As in ría ’h’, and Zn, Cd, Cr in ría ’m’) near wastewater treatment plants, spill zones, and major roads,^60^ suggesting the ongoing influence of anthropogenic impacts. In contrast, ría ’d’ (Ría O Burgo), previously considered one of the most polluted rías in Europe, showed substantial PTE decreases across most sites (Figure 3 and Figures S12–S19).

Elemental correlations and PCA identified two major groupings: Al–Fe and Cu–Cr–Ni–Zn-Hg, while Cd and As showed independent patterns (Figure S20). PMF further resolved four pollution sources: a potential natural source dominated by Al and Fe,^61,62^ a combined natural and industrial source for Ni and Cr^63–65^ (with Cr shifting toward natural sources in 2021, possibly due to increased runoff from land degradation^66^), and a consistent grouping of Zn, As, Cd, and Hg, which may reflect industrial, agricultural, and mining activities.^67–69^ Cu, initially separate in 1990 and 2001–2007, evolved from potential agricultural use, especially in vineyards,^70,71^ to association with Zn in 2021, suggesting modern agrochemicals or antifouling paint inputs^72,73^ (Figure 4).

### 4.3 Underlying Drivers of Long-Term PTE Trends

Several factors likely contributed to the observed trends. Notably, the decrease in PTE levels in *Fucus* spp. aligns with the implementation of significant environmental policies and international agreements, including the Urban Waste Water Treatment Directive,^28^ the OSPAR Convention,^74^ the Water Framework Directive,^75^ and the Marine Strategy Framework Directive.^24^ These initiatives collectively improved wastewater treatment systems and limited metal discharges into marine ecosystems. Specifically, the Urban Waste Water Treatment Directive played a crucial role by driving the construction of treatment plants and modernization of existing facilities, with 21 new treatment plants by 2000, 34 by 2010, and 11 more by 2020, in the study area.^60,76^ Complementary regulations such as the Integrated Pollution Prevention and Control Directive,^77^ the Industrial Emissions Directive,^78^ and the Minamata Convention on Mercury^79^ targeted metal emissions, while waste valorization practices may have reduced direct metal releases (Figure 5).^80,81^

**Figure 5:**
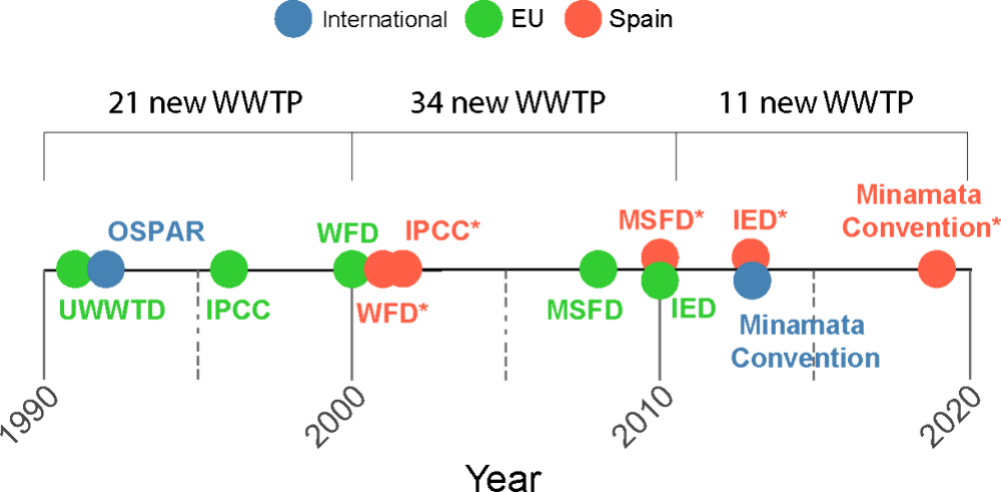
Timeline of key environmental regulations impacting marine environment. Asterisks (∗) denote Spanish legislation implemented in response to prior international or European agreements. UWWTD: Urban Waste Water Treatment Directive. OSPAR: Oslo and Paris Convention for the Protection of the Marine Environment of the North-East Atlantic. IPCC: Integrated Pollution Prevention and Control Directive. WFD: Water Framework Directive. MSFD: EU Marine Strategy Framework Directive. IED: Industrial Emissions Directive. WWTP: Wastewater Treatment Plant (numbers indicate newly constructed WWTPs discharging into the rías within the study area).^60^

These measures may have collectively lowered anthropogenic metal inputs in marine ecosystems, aligning with both reported emission declines^82^ and the PTE levels reductions observed in this study. PMF analysis confirmed this link, attributing most PTEs (excluding Al and Fe, and partially Ni and Cr) to human activities. Additionally, the detected PTE increases near wastewater treatment outfalls suggest that *Fucus* spp. may effectively capture anthropogenic pollution.

The observed trends may reflect not only decreasing metal concentrations in the marine environment but also reduced metal bioavailability, supported by weak correlations between seawater and algae PTE levels.^83–85^ Environmental drivers, like ocean acidification, temperature, and salinity shifts can alter PTEs speciation,^86,87^ while organic matter may affect bioavailability through chelation and particulate adsorption.^88,89^ However, the exact mechanisms require further study. Sediment dynamics further complicate the interpretation, as elevated Al and Fe, which are established sediment tracers, have increased in 2021 compared to 1990, suggesting higher sediment influence. Since Al correlated positively with Cr, Zn, and Hg, the measured declines in these metals may underestimate true reductions in bioavailable fractions, as sediment inputs likely masked anthropogenic decreases.

Additionally, physiological traits may influence PTE concentrations in *Fucus.* For instance, faster growth rates and nutrient limitation can increase metal uptake,^90^ while variations in cell wall polysaccharide composition may affect metal binding through their functional groups.^91^ Differences in the abundance of physodes, vesicles that contain phlorotannins with known metal-binding properties, may also play a role.^92,93^ Epiphytic microbiomes^94^ and potential genetic or epigenetic adaptations,^95^ add further complexity. Despite considerable efforts, the specific effects and temporal dynamics of these physiological factors remain unclear. However, preliminary, unpublished data from the algae samples in this study suggest temporal variations in cell wall composition, which may partly explain the observed trends.

This study also revealed increasing concentrations of Al, Fe, and As. Al and Fe, considered low-toxicity sediment tracers, likely reflect greater sediment contributions,^47^ as supported by correlations, PCA, and PMF analyses. In contrast, the increase in As appears to result from a combination of factors. Its positive correlation with Al (r = 0.23) and Fe (r = 0.11) suggests a sediment influence, yet this does not explain why similarly correlated elements such as Cr, Zn, Hg did not show comparable trends. The distinct behavior of As, evident in its PCA separation and its higher concentrations in *F. vesiculosus* and *F. spiralis* relative to *F. ceranoides*, suggests additional dynamics.

Environmental variables such as temperature, salinity, and organic matter likely modulate As mobility and bioavailability.^58,96^ While reported anthropogenic As emissions have declined in the EU,^97,98^ rising concentrations in this study and in other organisms and environmental compartments,^99,100^ though scarcely discussed, point to overlooked emerging sources such as groundwater discharges.^101,102^ The complexity and persistence of As in coastal ecosystems underscore the need for further investigation into its sources and accumulation pathways in coastal ecosystems.

### 4.4 Limitations and Implications

This study has several limitations. Variability in sediment input, reflected in fluctuating Fe and Al concentrations, complicates the interpretation of temporal trends, particularly for As, which showed associations with these elements while increasing significantly. The complex interplay of pollution with physicochemical and biological factors also makes it challenging to fully attribute changes in PTE concentrations to reduced pollution following environmental regulations. Consequently, the combined effects of factors affecting PTE concentrations in algae remain poorly understood and need further investigation. Although this may appear to limit the utility of *Fucus* as a biomonitor, it actually highlights a key strength: *Fucus* spp. concentrates bioavailable metal fractions over time, capturing contamination dynamics that water or sediment samples cannot, highlighting the importance of continued seaweed monitoring for assessing coastal metal pollution.

Taxonomic challenges, such as distinguishing *F. spiralis* from *F. vesiculosus*, and the lack of overlapping sites for all three species, limited interspecific comparisons of bioconcentration capacity. Although the toxic effects of PTEs on algae are documented,^103,104^ our understanding of the underlying mechanisms is still limited,^105,106^ particularly regarding subcellular distribution and metal speciation.^51^ While highly toxic forms like arsenite and methylmercury are believed to be less prevalent in brown algae,^107–109^ their specific contributions and risks remain unclear. Some *Fucus* populations may have evolved mechanisms to limit PTE uptake,^110^ but climate-related stressors like ocean acidification may increase their vulnerability, as seen in recent declines linked to heatwaves and increased wave action.^111,112^ Understanding the combined effects of PTE exposure and environmental stressors is critical for predicting the future of these species.

Despite these challenges, our results show clear long-term changes in PTE concentrations in *Fucus* spp., consistent with declining contamination inputs across the EU. The consistency of patterns across multiple metals supports our conclusions and reinforces the suitability of *Fucus* as a biomonitor at decadal scales. These findings are ecologically relevant, given the importance of *Fucus* spp. in coastal ecosystems, and provide reference values for PTE concentrations in the study area. This study underscores the importance of sustained, long-term monitoring, as such trends would remain unnoticed without multidecade observations.

## Supplementary Material





## Notes

Additional information for Linear Mixed Models (LMM). Table S1 (Number of sampling sites per year and species). Table S2 (Quality analysis results). Table S3 (Median PTE concentrations per year and species, and significant interannual Dunn’s test results). Table S5 (Bioconcentration factors and sediment contribution). Table S6 (Summary of key outputs from LMMs). Table S7 (Percentage of variability explained by each factor in PMF models). Figures S1–S9 (Overview of PTEs concentrations on the sampling sites). Figures S10–S11 (Overview of PTEs concentrations over time). Figures S12–S19 (Maps of percentage changes in PTES concentrations). Figure S20 (Correlation matrix and PCA). (PDF)
Table S4 (Detailed PTE concentrations per sampling site and sampling campaign) (XLSX)
