# Supplementary material for: Three Decades of Change in Potentially Toxic Elements in Brown Algae in the Northeast Atlantic Ocean
Source: Environ Sci Technol. 2025 May 22;59(21):10476–87. doi: 10.1021/acs.est.4c14013 (PMC12138972; doi:10.1021/acs.est.4c14013)
Supplement: Supplementary file 1 [file es4c14013_si_001.pdf]

Three Decades of Change in Potentially Toxic Elements in Brown Algae in the Northeast Atlantic Ocean

Carme Pacín[1,2*], J. Ángel Fernández[1], Mercedes Conde-Amboage[3], Massimo Lazzari[2], Rita García-Seoane[4,5], Inés G. Viana[4], Zulema Varela[1], Carlos Real[6], Rubén Villares[6], Jesús R. Aboal[1]

1. CRETUS Centre, Department of Functional Biology, Ecology Unit, Universidade de Santiago de Compostela, Santiago de Compostela, 15782, Spain
2. CIQUS Centre, Department of Physical Chemistry, Universidade de Santiago de Compostela, Santiago de Compostela, 15782, Spain
3. Department of Statistics, Mathematical Analysis and Optimization, Universidade de Santiago de Compostela, Santiago de Compostela, 15782, Spain
4. Instituto Español de Oceanografía (IEO-CSIC), Centro Oceanográfico de A Coruña, A Coruña, 15001, Spain
5. Department of Earth Sciences, University of Hawaii at Mānoa, 1680 East-West Road, POST 719B, Honolulu, HI, 96822, United States
6. Department of Functional Biology, Ecology Unit, Universidade de Santiago de Compostela, Escola Politécnica Superior de Enxeñaría Lugo, 27002, Spain

* Corresponding author. Email: mcarme.pacin@usc.es

Number of pages: 36. Number of tables: 7. Number of figures: 20

Supporting Information includes:

- Additional information for Linear Mixed Models (LMM)
- Supplementary tables. Table S1 (Number of sampling sites per year and species), Table S2 (Quality analysis results), Table S3 Median PTE concentrations per year and species, and significant interannual Dunn's test results), Table S4 (Detailed PTE concentrations per sampling site and sampling campaign), Table S5 (Bioconcentration factors and sediment contribution), Table S6 (Summary of key outputs from LMMs), and Table S7 (Percentage of variability explained by each factor in PMF models).
- Supplementary figures. Figs. S1-S9 (Overview of PTEs concentrations on the sampling sites), Figs. S10- S11 (Overview of PTEs concentrations over time), Figs. S12-S19 (Maps of percentage changes in PTES concentrations), S20 (correlation matrix and PCA).

Supporting information_2_TableS4 includes Table S4 which consists in an excel file containing sampling sites information including Coordinates (WGS84), Ría, Species, Year of sampling, and Potentially Toxic Elements (PTEs) concentrations.

Additional information for Linear Mixed Models (LMM)

All data were first explored for outliers, normality, homogeneity of variance and collinearity. Logarithm transformations were applied to obtain a better approximation to a normal distribution.

The statistical significance of interannual trends in elements was assessed, with species and year of sampling as fixed factors, and ría as random factor, fitting several models:

Model 1. Element concentration ~ Year

Model 2. Element concentration ~ Year + (1 | ría)

Model 3. Element concentration ~ Year + (Year | ría)

Model 4. Element concentration ~ Year + Species + (1| ría)

Model 5. Element concentration ~ Year + Species + (Year| ría)

The different models were compared using the Bayesian Information Criterion (BIC) and the Akaike Information Criterion (AIC). From each model, we obtained fitted values, residuals, R^2, p values for F-statistics and goodness of fit by an analysis of variance (ANOVA). Standard residual validation techniques (q–q plot, residual histograms and scatterplots of predictors vs. residuals) were applied to validate the final model. Main outputs of the selected models can be found in Table S4, with p-values corrected using the Benjamini-Hochberg (BH) method

Supplementary Tables

Table S1. Number of sampling sites per year and species.

	1990	2001	2003	2005	2007	2021
Fucus ceranoides	79	14	14	14	14	58
Fucus vesicuosus - F. spiralis	48	25	34	35	35	76

Table S2. Quality analysis results in terms of limit of quantification (LOQ) expressed in µg l^{-1} for all elements except Hg (ng g^{-1}), sample size, Relative Percent Differences (RPD, %), and recovery from reference materials (%). The recovery is shown only for elements with certified concentrations in the reference material.

	**Sample size**	LOQ	RPD	Recovery
Al	16	29.13	5.3	-
Cr	16	0.97	8.9	-
Fe	16	17.72	3.1	-
Ni	16	0.49	3.3	-
Cu	16	3.11	4.1	89
Zn	16	7.31	3.3	96
As	16	1.48	2.5	109
Cd	16	0.55	3.4	99
Hg	31	3.59	2.7	97

Table S3. Median concentrations of Al, Cr, Fe, Ni, Cu, Zn, As, and Cd (µg g^{-1}, dry weight), and Hg (ng g^{-1}, dry weight) in *Fucus* spp. collected from NW Spain (1990-2021). 'Year' indicates sampling year, and 'All' combines all years. *F.v-F.s* refers to *F. vesiculosus* and *F. spiralis*, *F.c* to *F. ceranoides,* and *Fucus* spp. represents these species combined. COD (coefficient of dispersion) represents the median absolute deviation divided by the median (%). The 'Dunn-test' column identified significant interannual variations through Dunn's post-hoc tests with Benjamini-Hochberg correction, following significant Kruskal-Wallis results (α = 0.001) across all *Fucus* spp. Significant differences (p<0.05) are indicated by distinct lowercase letters.

Element	Year	Median (*F.v-F.s*)	Median (*F.c*)	Median (*Fucus* spp.)	COD (*Fucus* spp.)	Dunn's test (*Fucus* spp.)
**Al n=446**	1990	80.3	143	124	44.1	a
	2001	134	585	263	66.0	a
	2003	51.3	642	82.3	86.4	a
	2005	52.3	617	147	94.1	a
	2007	45.8	559	75.3	87.8	a
	2021	413	1515	917	80.6	b
	All	90.5	502	180	85.0	
**Cr n=446**	1990	8.26	10.1	8.64	71.3	a
	2001	3.64	7.34	4.41	48.2	ab
	2003	2.48	9.64	3.15	60.4	b
	2005	3.13	11.1	6.54	60.3	ab
	2007	1.62	8.88	2.30	53.4	b
	2021	0.67	1.62	1.14	78.1	c
	All	2.23	7.10	3.16	82.0	
**Fe n=446**	1990	204	315	277	32.1	a
	2001	172	499	295	52.3	ab
	2003	111	573	172	58.1	a
	2005	123	650	197	73.4	a
	2007	101	565	131	58.8	a
	2021	257	994	651	70.8	b
	All	164	506	295	65.9	
**Ni n=446**	1990	9.84	11.0	10.6	41.7	a
	2001	5.66	7.46	5.92	25.9	b
	2003	3.33	7.80	4.31	41.5	b
	2005	3.53	9.55	4.72	42.9	b
	2007	4.03	8.25	4.63	42.6	b
	2021	1.39	2.53	1.65	38.4	c

	All	3.52	7.57	5.00	60.6	
**Cu n=446**	1990	47.8	47.4	47.4	60.7	a
	2001	11.4	13.7	12.0	54.5	bd
	2003	3.39	6.48	4.89	52.6	c
	2005	8.68	13.5	10.4	50.8	d
	2007	17.9	21.9	18.3	46.7	ab
	2021	2.47	4.75	3.39	52.5	c
	All	7.68	14.9	10.1	75.1	
**Zn n=446**	1990	45.8	55.1	50.4	39.0	a
	2001	46.2	71.3	53.2	29.5	a
	2003	39.1	49.9	44.3	24.3	ab
	2005	38.5	52.5	40.6	19.4	ab
	2007	38.1	77.8	41.3	30.3	b
	2021	31.7	45.8	37.4	36.2	b
	All	38.9	52.8	43.4	33.3	
**As n=446**	1990	40.7	35.2	37.6	25.8	a
	2001	36.8	30.0	34.1	16.4	a
	2003	33.7	23.7	31.7	22.0	ab
	2005	28.5	23.0	27.9	13.1	cb
	2007	28.3	19.5	25.3	14.6	c
	2021	61.8	46.4	55.8	29.1	d
	All	37.7	34.9	36.0	28.7	
**Cd n=446**	1990	0.84	0.85	0.8	30.4	a
	2001	0.58	0.86	0.7	25.8	ab
	2003	0.66	0.66	0.7	34.1	bc
	2005	0.79	0.56	0.7	24.3	ab
	2007	0.65	0.66	0.6	25.5	cb
	2021	0.50	0.51	0.5	26.1	c
	All	0.64	0.68	0.66	32.7	
**Hg n=425**	1990	60.8	69.2	64.6	39.0	a
	2001	24.4	20.4	24.1	25.1	b
	2003	17.4	19.5	18.1	24.9	b
	2005	16.1	21.7	17.1	29.8	b
	2007	16.4	22.5	18.2	30.4	b
	2021	16.4	18.1	17.5	26.3	b
	All	20.3	29.7	22.5	41.3	

Table S4. Excel file (provided in Supplementary material 2) containing sampling sites information including Coordinates (WGS84), Ría, Species, Year of sampling, and Potential Toxic Elements (PTEs) concentrations for each sample of Fucus collected in NW Spain from 1990 to 2021. Element concentrations are expressed in µg g-1, except for Hg in ng g -1. Additionally, the Relative Standard Deviation (RSD, expressed as a percentage) is included.

Table S5. Analysis of: (1) bioconcentration factors (BCF) for Potentially Toxic Elements (PTEs) in *Fucus* spp. (2021 data) relative to seawater (2023 data), and (2) sediment-derived contributions (%) to algal PTE concentrations (year-matched samples). Calculations follow Equations (1) and (2) (Material and Methods section).

	**Year**	**Al**	**Cr**	**Cu**	**Fe**	**Ni**	**Zn**	**Cd**	**Hg**
**BCF seawater**	2021/ 2023	39.1	10.4	6.26	64.5	2.48	0.047	20.3	-
**Sediment contrib.**	1990		0.43	0.095	11.4	0.25	0.37	-	
	2001		-	-	112	11.5	4.36	-	
	2003		-	-	99.1	6.06	3.52	-	
	2005		7.54	4.56	83.4	6.06	5.41	0.67	17.2
	2007		6.24	1.96	60.4	4.26	2.28	0.61	8.16
	All		2.18	0.23	54.0	3.54	1.33	0.66	15.0

Table S6. Summary of key outputs from linear mixed models assessing temporal trends of Potential Toxic Elements (PTEs). The "Model" column indicates the selected model (see combinations tested in Additional information for Linear Mixed Models (LMM) at this file). "R^2 marg" and "R^2 cond" represent marginal and conditional R^2, respectively. Columns include estimates for "Year" and "Species", residual variance, and p-values corrected using the Benjamini-Hochberg method for both "Year" and "Species".

PTE	Model	Estimate (Year)	Estimate (Species)	Residual Variance	R^2 marg	R^2 cond	p-value year	p-value species
**Al**	Model 4	0.063	-1.532	1.212	0.395	0.513	4.515E-37	1.085E-35
**Cr**	Model 5	-0.073	-0.681	0.922	0.416	0.601	1.735E-06	2.238E-11
**Fe**	Model 4	0.024	-1.101	0.649	0.305	0.411	1.068E-12	4.309E-35
**Ni**	Model 5	-0.051	-0.371	0.342	0.469	0.635	4.135E-09	1.762E-09
**Cu**	Model 5	-0.085	-0.557	0.974	0.480	0.609	9.823E-08	4.356E-08
**Zn**	Model 5	-0.011	-0.402	0.210	0.138	0.550	9.866E-04	2.947E-16
**As**	Model 5	0.012	0.129	0.204	0.103	0.212	1.837E-03	4.486E-03
**Cd**	Model 2	-0.015	-	0.250	0.093	0.320	4.788E-13	-
**Hg**	Model 5	-0.037	-0.267	0.314	0.299	0.574	2.540E-07	6.998E-06

Table S7. Percentage of variability explained by each factor in the Positive matrix factorization models applied separately for the sampling dates 1990, 2001-2007, and 2021.

	Factor 1	Factor 2	Factor 3	Factor 4
**1990**	24.88	37.48	25.96	11.68
**2001-2007**	30.61	35.16	18.64	15.59
**2021**	38.34	25.16	19.66	16.84

Supplementary Figures

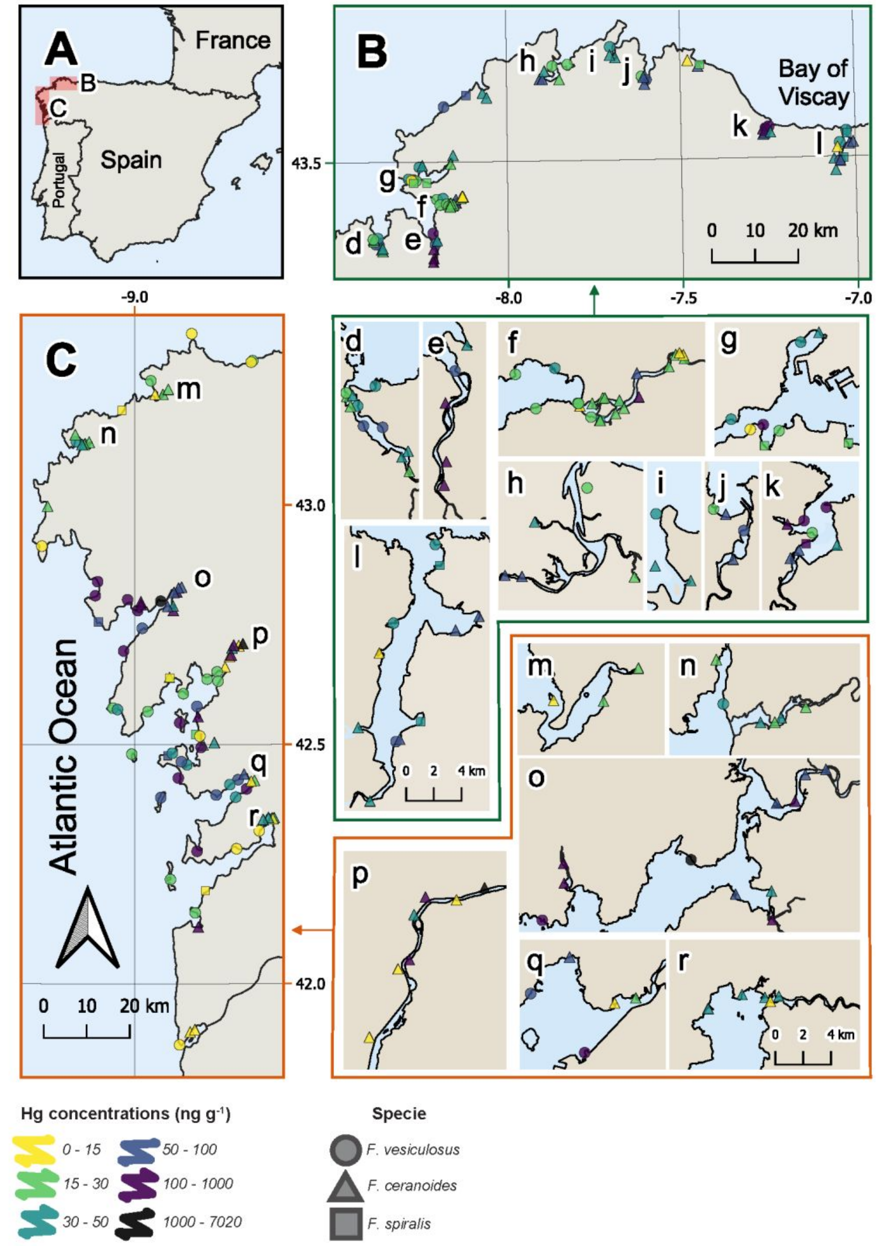

Fig. S1. Overview of Hg median concentrations (ng g^{-1}) in the sampling sites. Panels A-C display an overview of the region, with B and C showing the sampling sites. Panels d-l and m-r present detailed maps of sites that are densely clustered and difficult to distinguish in B and C, respectively. Different symbols represent the species sampled (*Fucus ceranoides, F. spiralis* and *F. vesiculosus*).

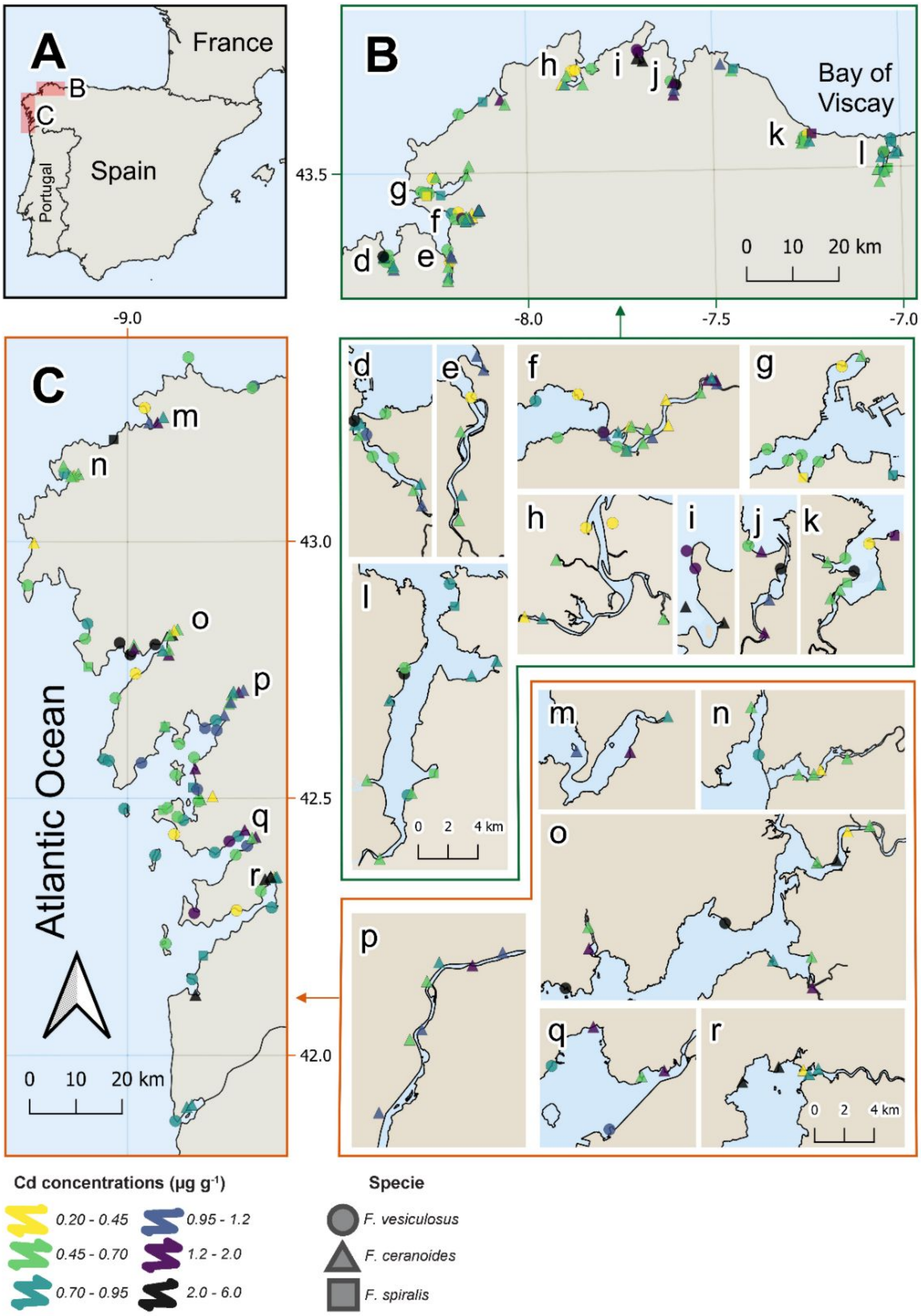

Fig. S2. Overview of Cd median concentrations (µg g^{-1}) in the sampling sites. Panels A-C display an overview of the region, with B and C showing the sampling sites. Panels d-l and m-r present detailed maps of sites that are densely clustered and difficult to distinguish in B and C, respectively. Different symbols represent the species sampled (*Fucus ceranoides, F. spiralis* and *F. vesiculosus*).

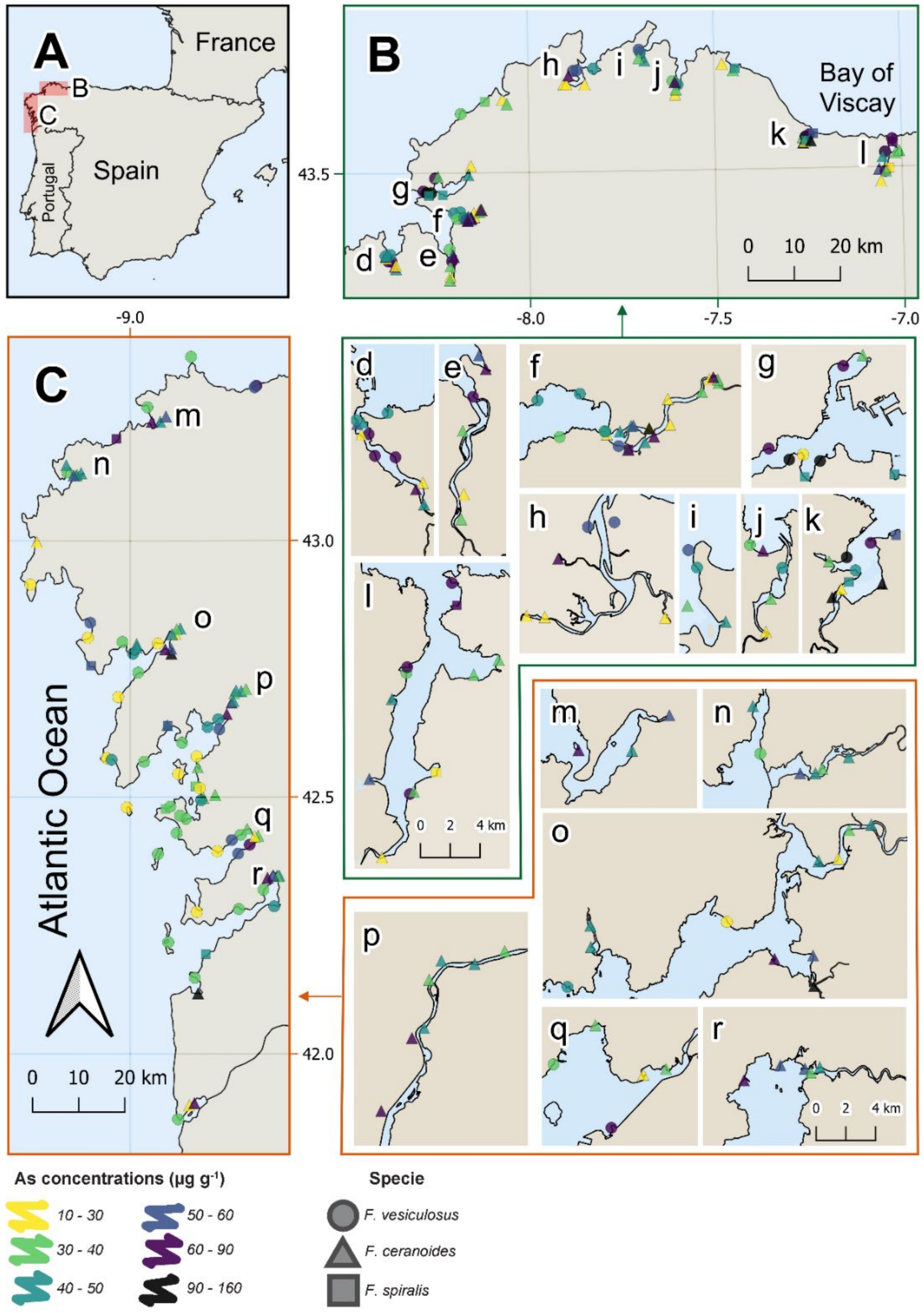

Fig. S3. Overview of As median concentrations (µg g^{-1}) in the sampling sites. Panels A-C display an overview of the region, with B and C showing the sampling sites. Panels d-l and m-r present detailed maps of sites that are densely clustered and difficult to distinguish in B and C, respectively. Different symbols represent the species sampled (*Fucus ceranoides, F. spiralis* and *F. vesiculosus*).

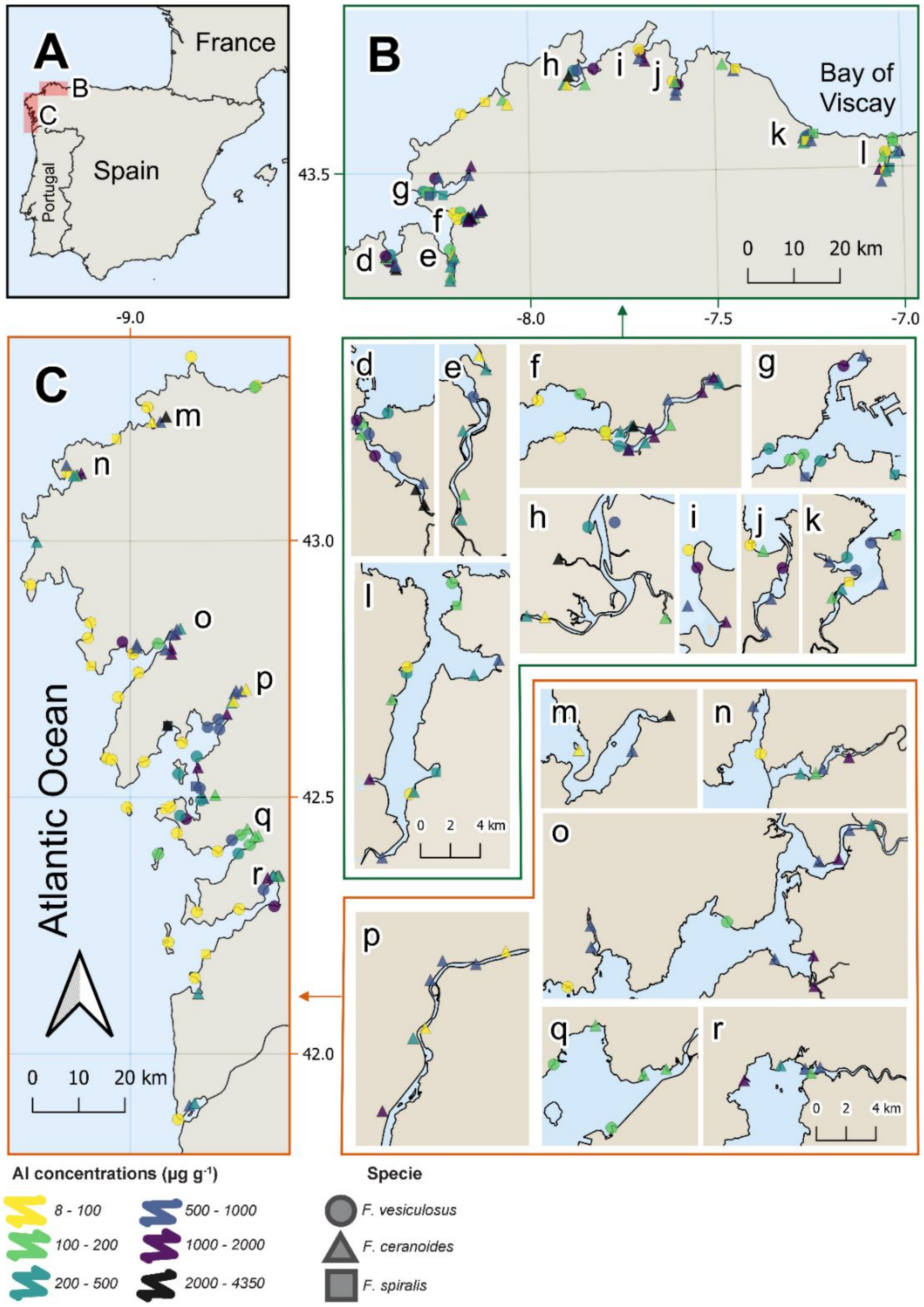

Fig. S4. Overview of Al median concentrations (µg g $^{-1}$) in the sampling sites. Panels A-C display an overview of the region, with B and C showing the sampling sites. Panels d-l and m-r present detailed maps of sites that are densely clustered and difficult to distinguish in B and C, respectively. Different symbols represent the species sampled (*Fucus ceranoides, F. spiralis* and *F. vesiculosus*).

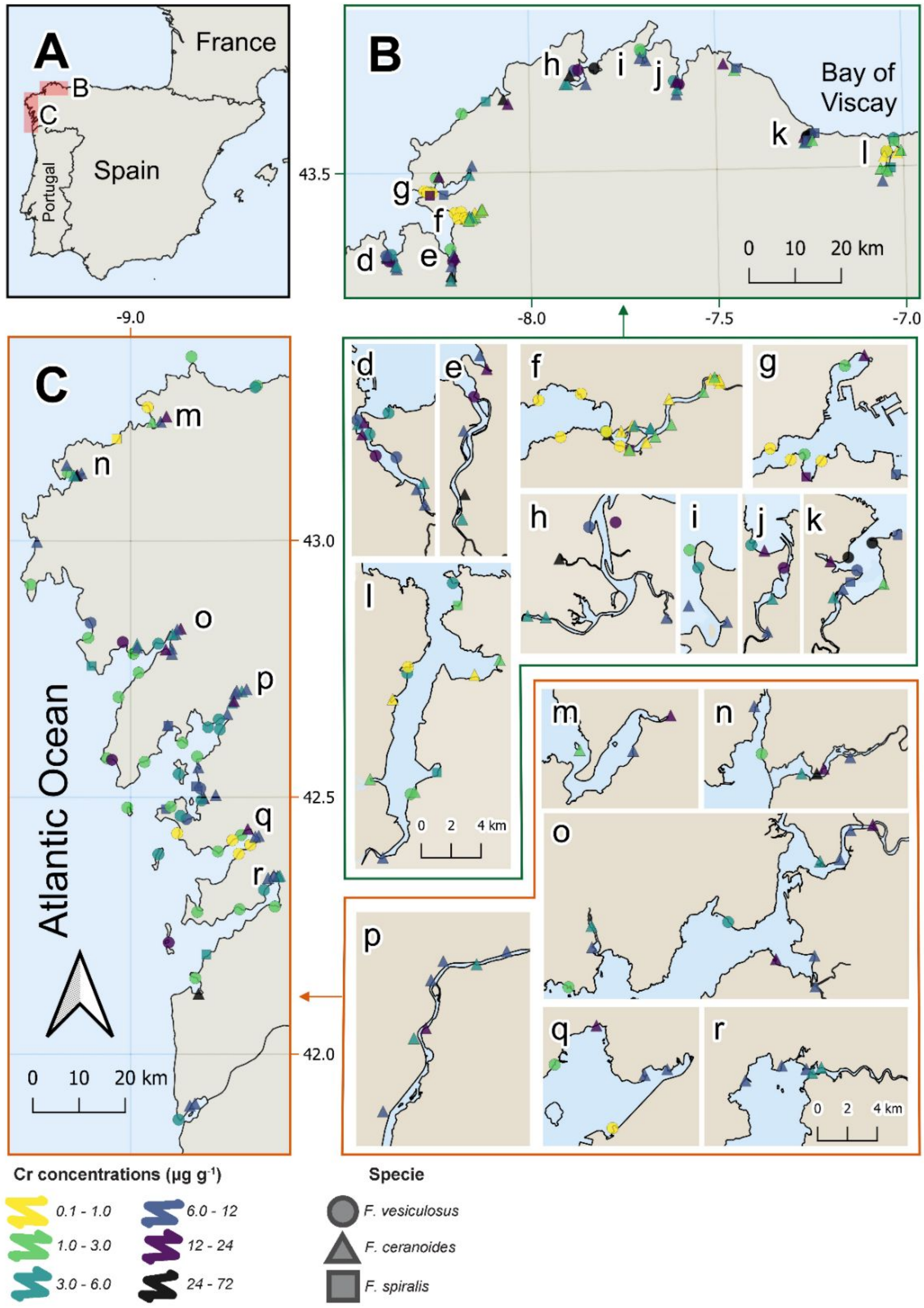

Fig. S5. Overview of Cr median concentrations (µg g^{-1}) in the sampling sites. Panels A-C display an overview of the region, with B and C showing the sampling sites. Panels d-l and m-r present detailed maps of sites that are densely clustered and difficult to distinguish in B and C, respectively. Different symbols represent the species sampled (*Fucus ceranoides, F. spiralis* and *F. vesiculosus*).

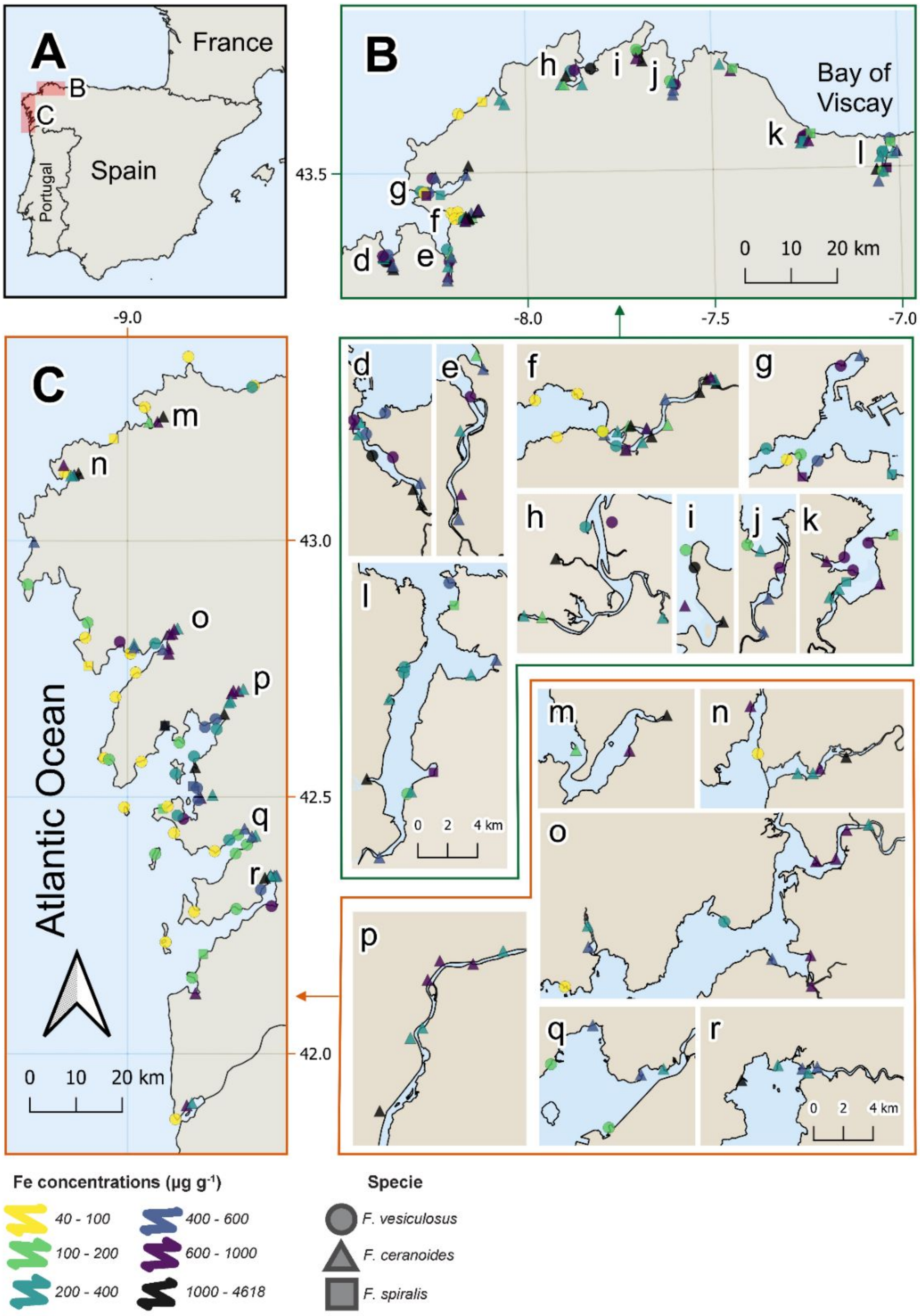

Fig. S6. Overview of Fe median concentrations (µg g $^{-1}$) in the sampling sites. Panels A-C display an overview of the region, with B and C showing the sampling sites. Panels d-l and m-r present detailed maps of sites that are densely clustered and difficult to distinguish in B and C, respectively. Different symbols represent the species sampled (*Fucus ceranoides, F. spiralis* and *F. vesiculosus*).

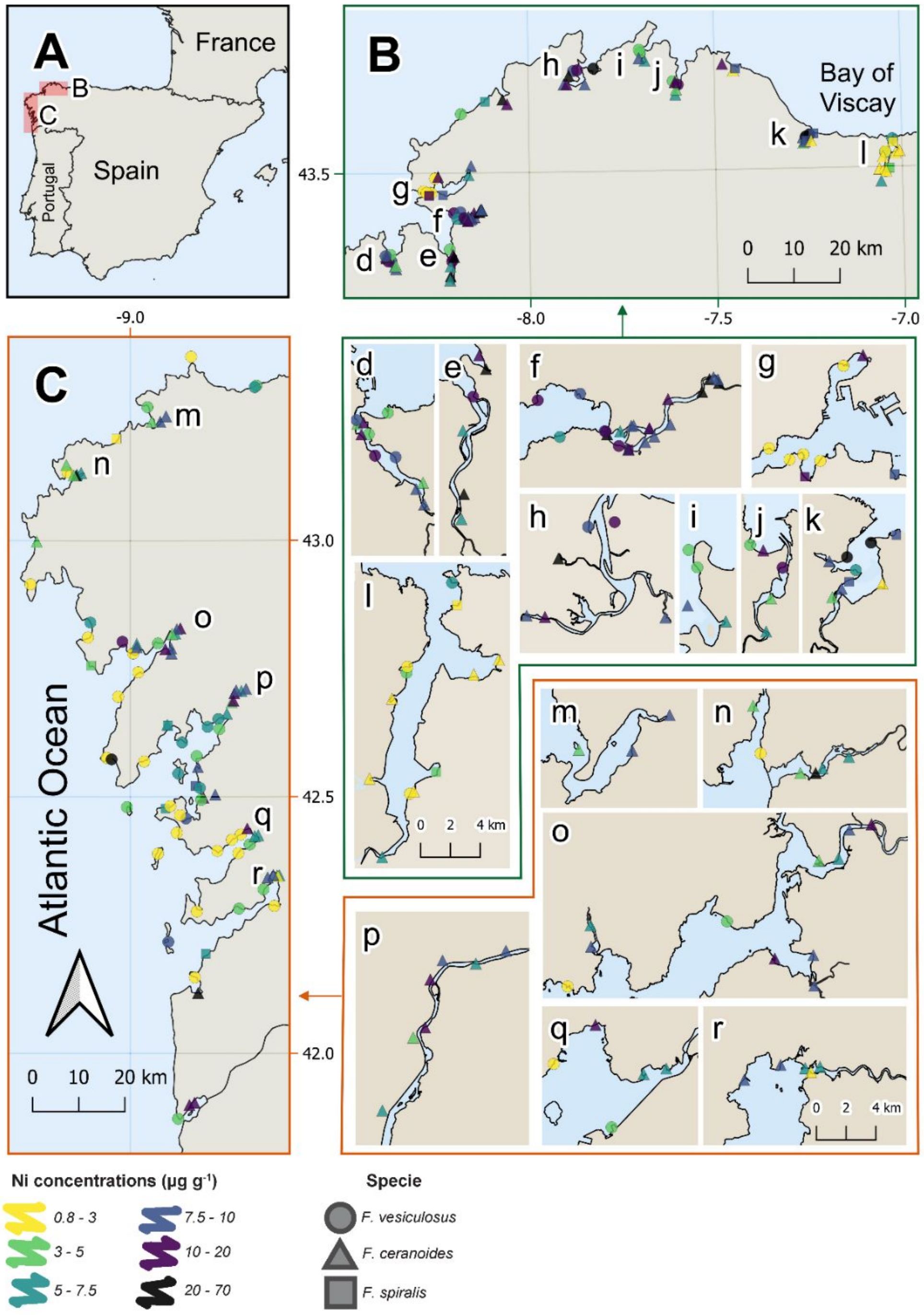

Fig. S7. Overview of Ni median concentrations (μg g^{-1}) in the sampling sites. Panels A-C display an overview of the region, with B and C showing the sampling sites. Panels d-l and m-r present detailed maps of sites that are densely clustered and difficult to distinguish in B and C, respectively. Different symbols represent the species sampled (*Fucus ceranoides, F. spiralis* and *F. vesiculosus*).

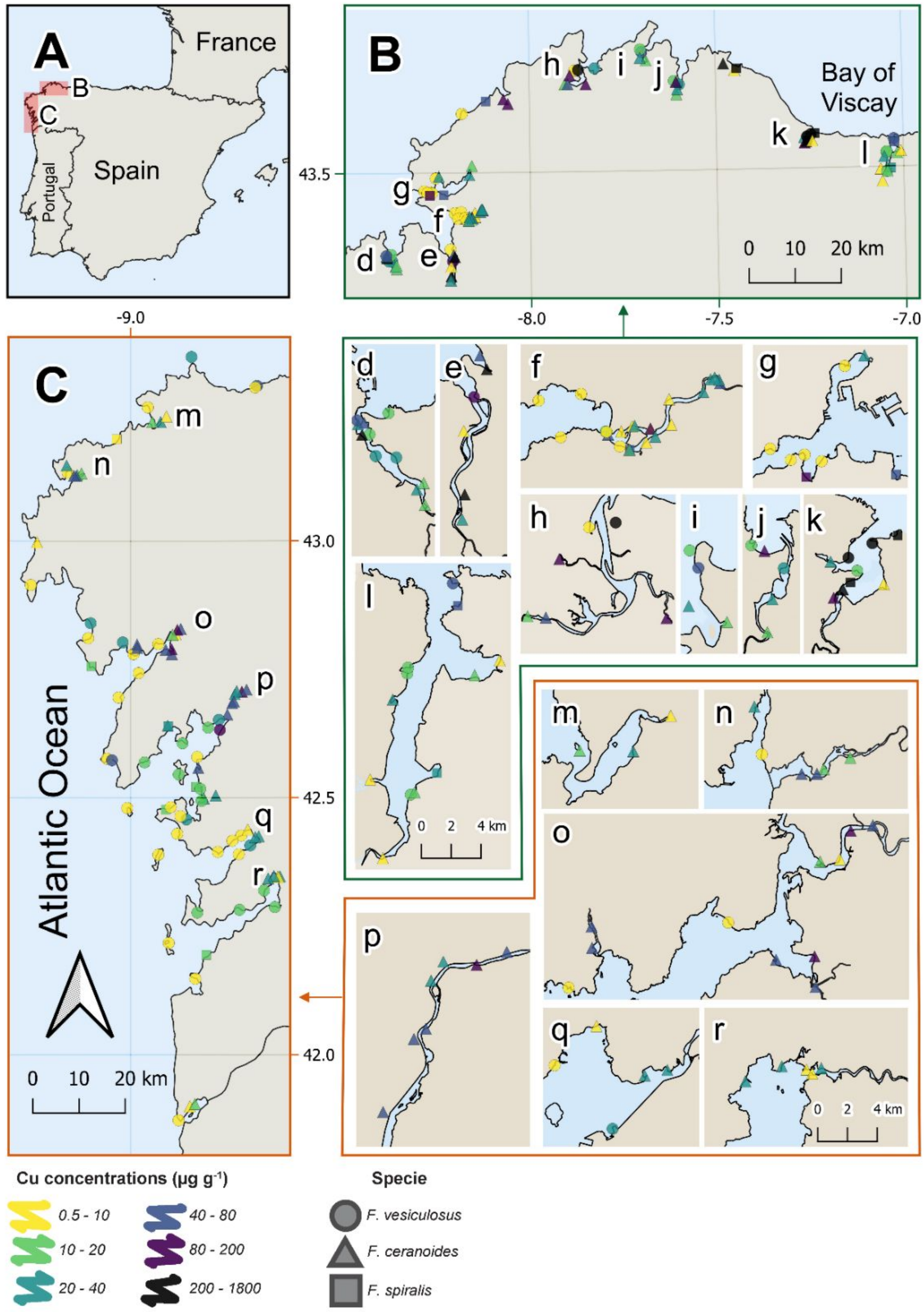

Fig. S8. Overview of Cu median concentrations (µg g^{-1}) in the sampling sites. Panels A-C display an overview of the region, with B and C showing the sampling sites. Panels d-l and m-r present detailed maps of sites that are densely clustered and difficult to distinguish in B and C, respectively. Different symbols represent the species sampled (*Fucus ceranoides, F. spiralis* and *F. vesiculosus*).

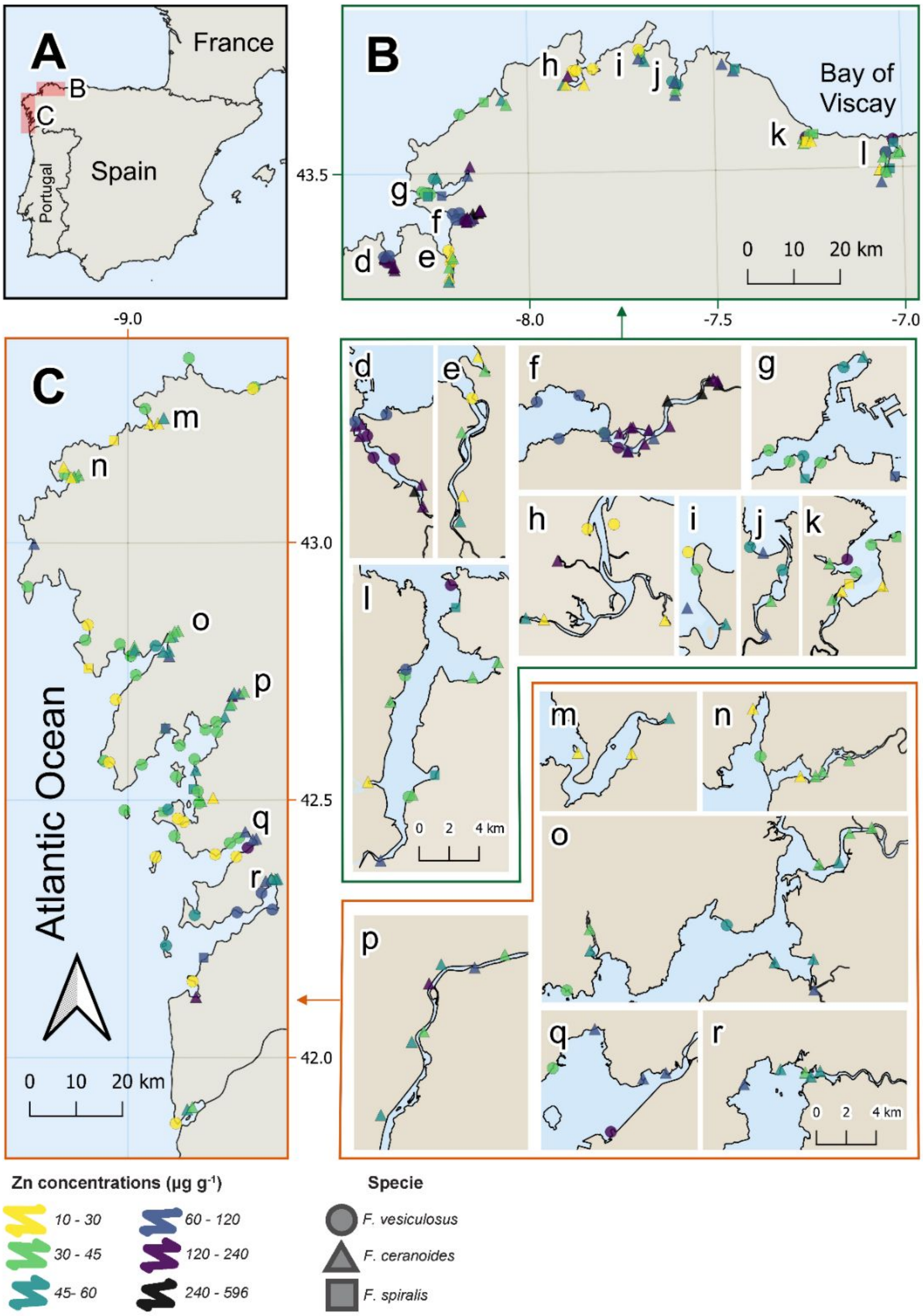

Fig. S9. Overview of Zn median concentrations (µg g^{-1}) in the sampling sites. Panels A-C display an overview of the region, with B and C showing the sampling sites. Panels d-l and m-r present detailed maps of sites that are densely clustered and difficult to distinguish in B and C, respectively. Different symbols represent the species sampled (*Fucus ceranoides, F. spiralis* and *F. vesiculosus*).

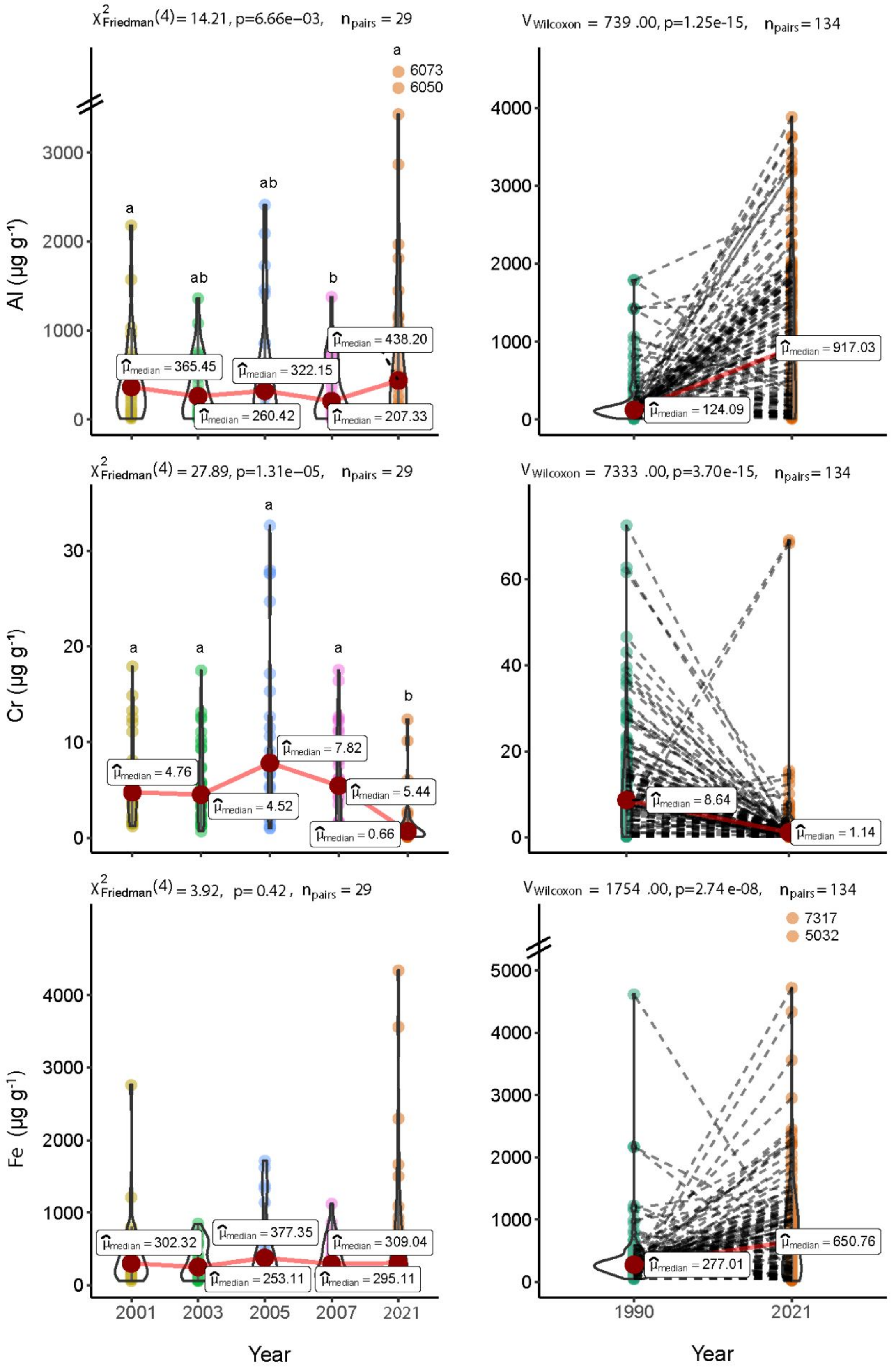

Fig. S10. Temporal trends of Al (µg g⁻¹), Cr (µg g⁻¹), and Fe (µg g⁻¹) concentrations in *Fucus* spp. Left panel: Repeated measures (2001-2021) analyzed by Friedman test with

Durbin-Conover post-hoc comparisons. Right panel: Paired 1990 vs 2021 comparisons (Wilcoxon test). Distinct lowercase letters indicate significant differences between years ($p<0.05$, no shared letters = significant).

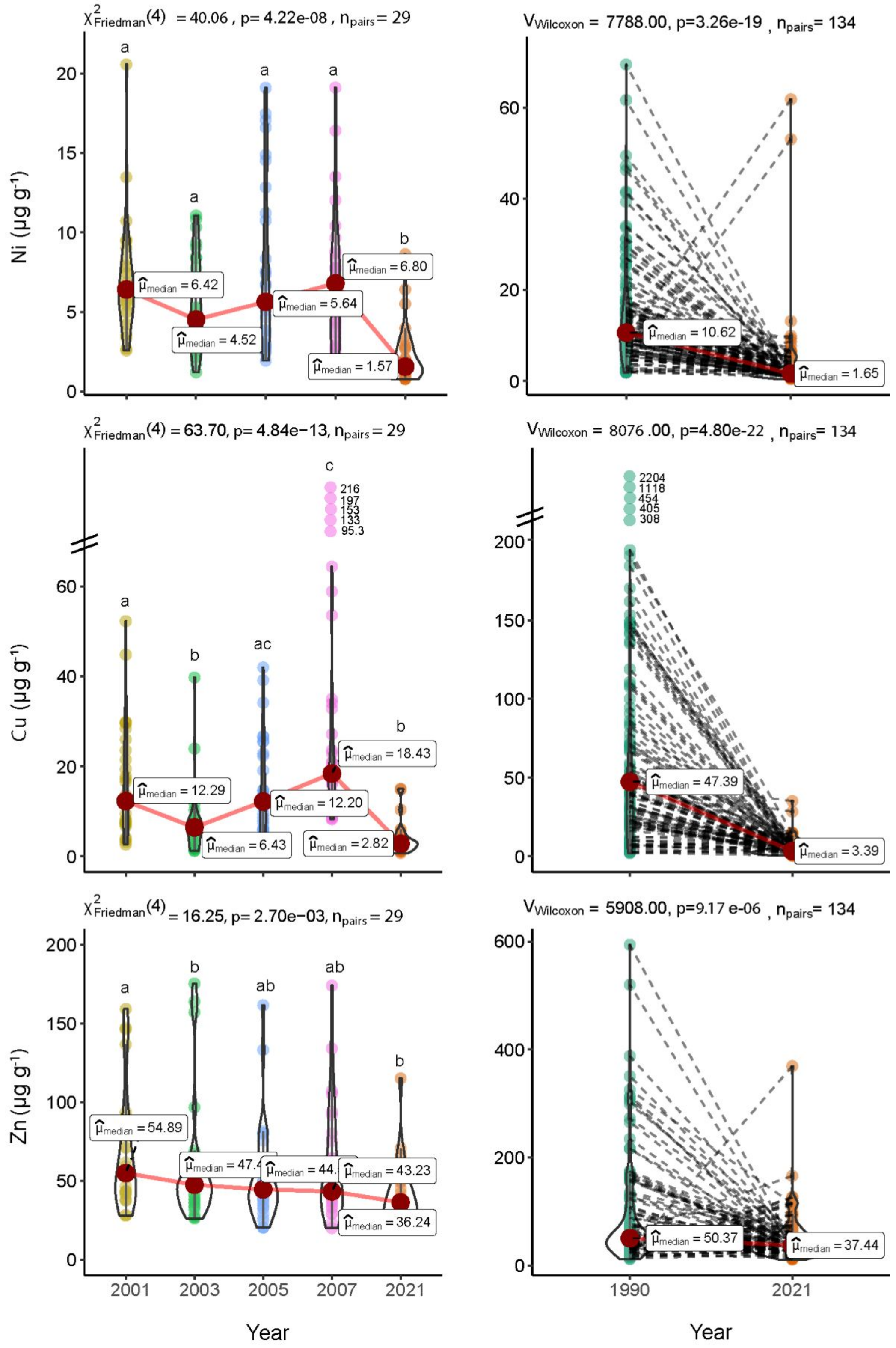

Fig. S11. Temporal trends of Ni (μg g^{-1}), Cu (μg g^{-1}), and Zn (μg g^{-1}) concentrations in *Fucus* spp. Left panel: Repeated measures (2001-2021) analyzed by Friedman test with Durbin-Conover post-hoc comparisons. Right panel: Paired 1990 vs 2021 comparisons (Wilcoxon test). Distinct lowercase letters indicate significant differences between years ($p<0.05$, no shared letters = significant).

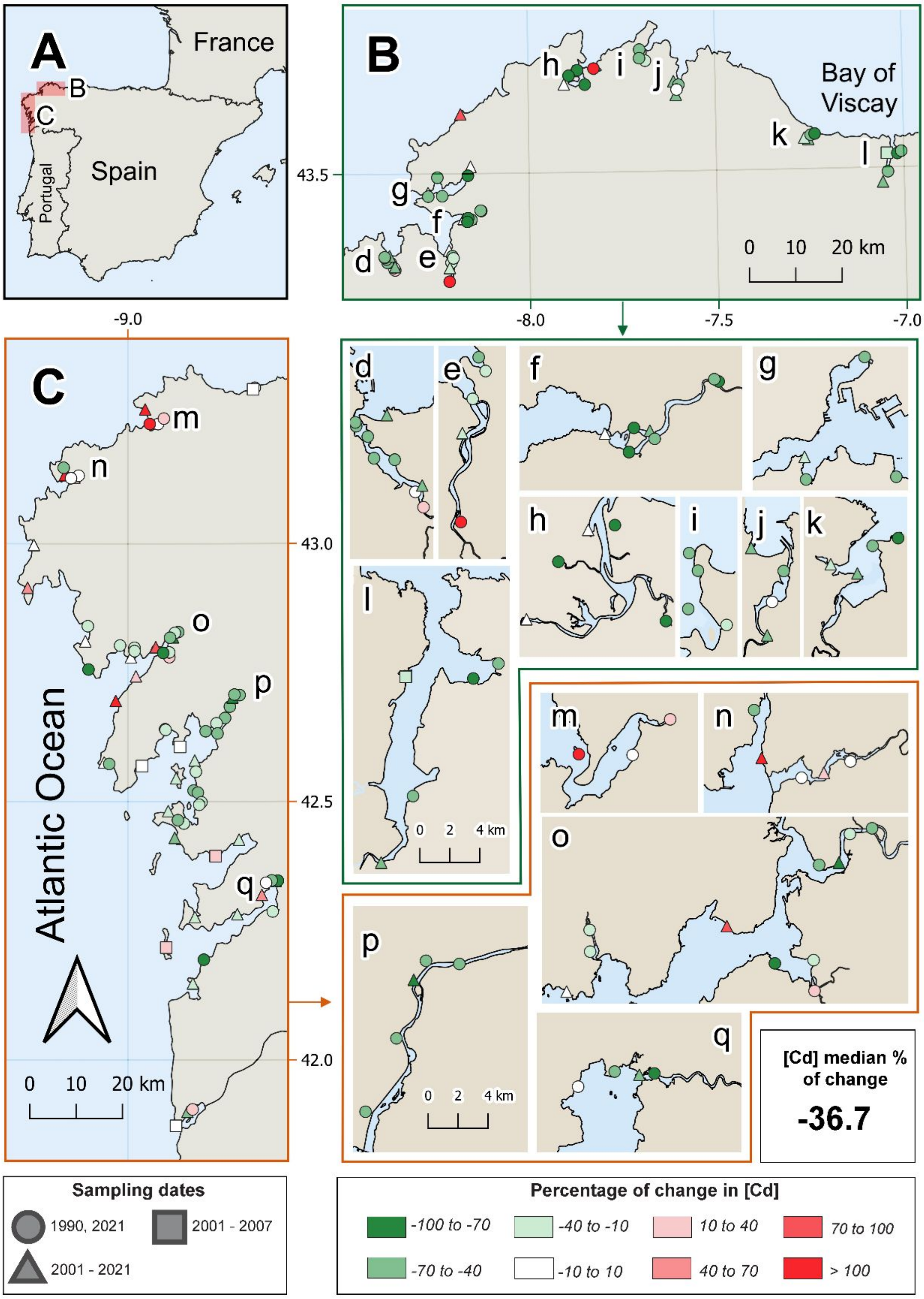

Fig. S12. Map of percentage changes in Cd concentrations over time. Panels A-C provide a regional overview, with B and C showing the differences between the final and initial

Cd concentrations (in %) at each sampling station. Panels d-l and m-q offer detailed views of sampling sites that are densely clustered and hard to distinguish in B and C, respectively. Different colors represent the percentage changes in Cd concentrations, while distinct symbols indicate the sampling dates: 1990 and 2021, 2001-2021, and 2001-2007. The total median percentage change, calculated as the median of the percentage changes across all sampling sites, is displayed below.

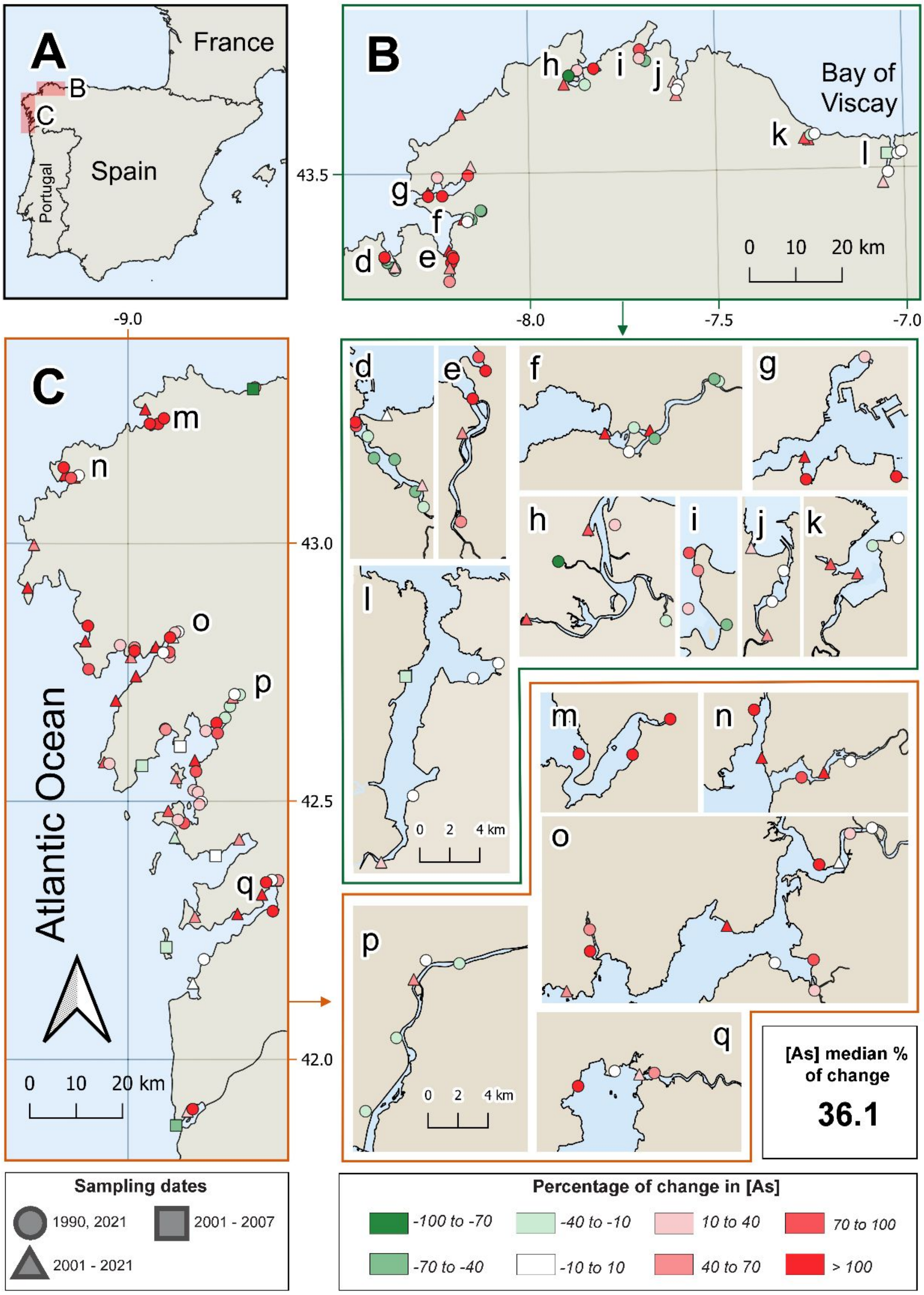

Fig. S13. Map of percentage changes in As concentrations over time. Panels A-C provide a regional overview, with B and C showing the differences between the final and initial As concentrations (in %) at each sampling station. Panels d-l and m-q offer detailed views

of sampling sites that are densely clustered and hard to distinguish in B and C, respectively. Different colors represent the percentage changes in As concentrations, while distinct symbols indicate the sampling dates: 1990 and 2021, 2001-2021, and 2001-2007. The total median percentage change, calculated as the median of the percentage changes across all sampling sites, is displayed below.

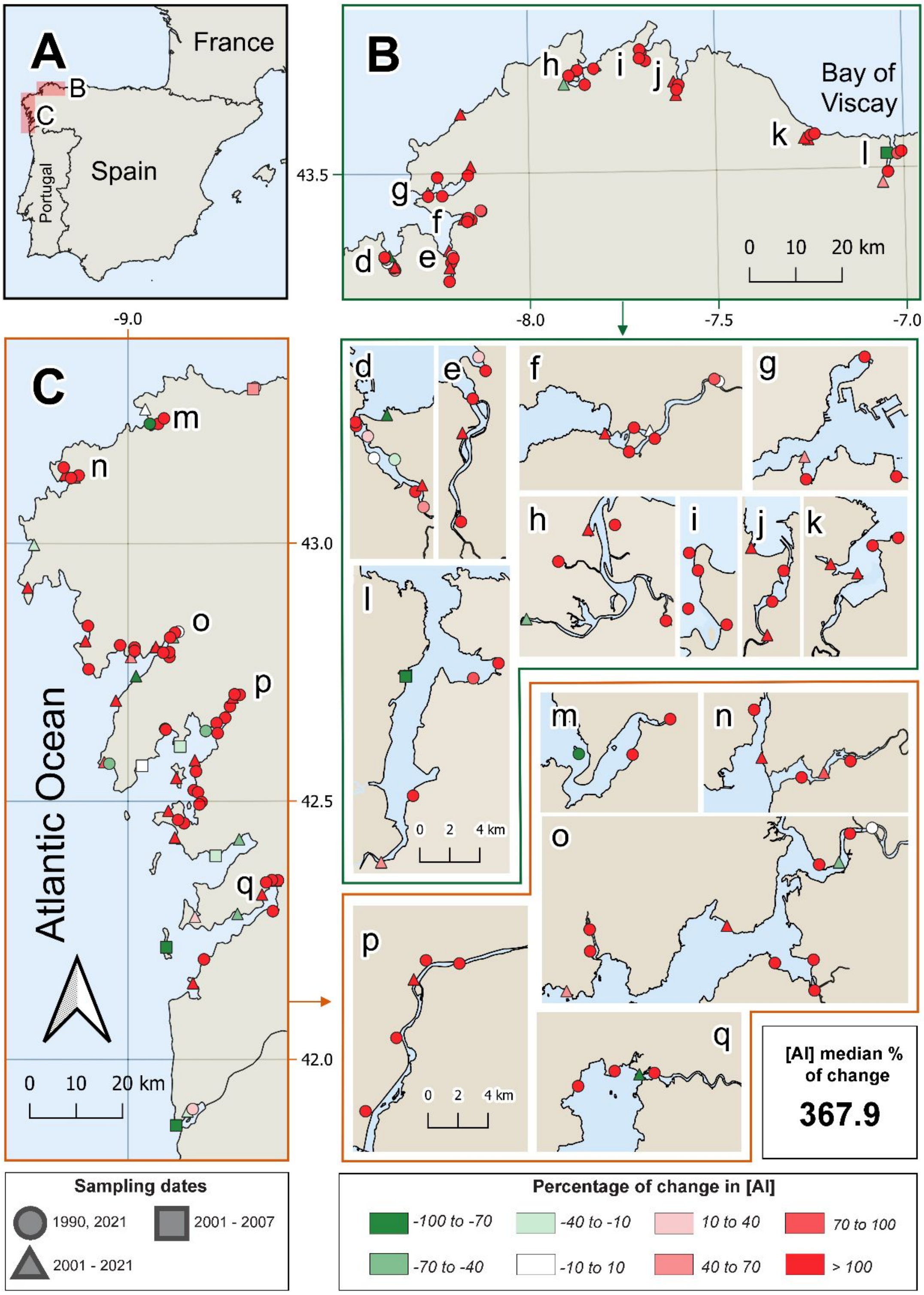

Fig. S14. Map of percentage changes in Al concentrations over time. Panels A-C provide a regional overview, with B and C showing the differences between the final and initial Al concentrations (in %) at each sampling site. Panels d-l and m-q offer detailed views

of stations that are densely clustered and hard to distinguish in B and C, respectively. Different colors represent the percentage changes in Al concentrations, while distinct symbols indicate the sampling dates: 1990 and 2021, 2001-2021, and 2001-2007. The total median percentage change, calculated as the median of the percentage changes across all stations, is displayed below.

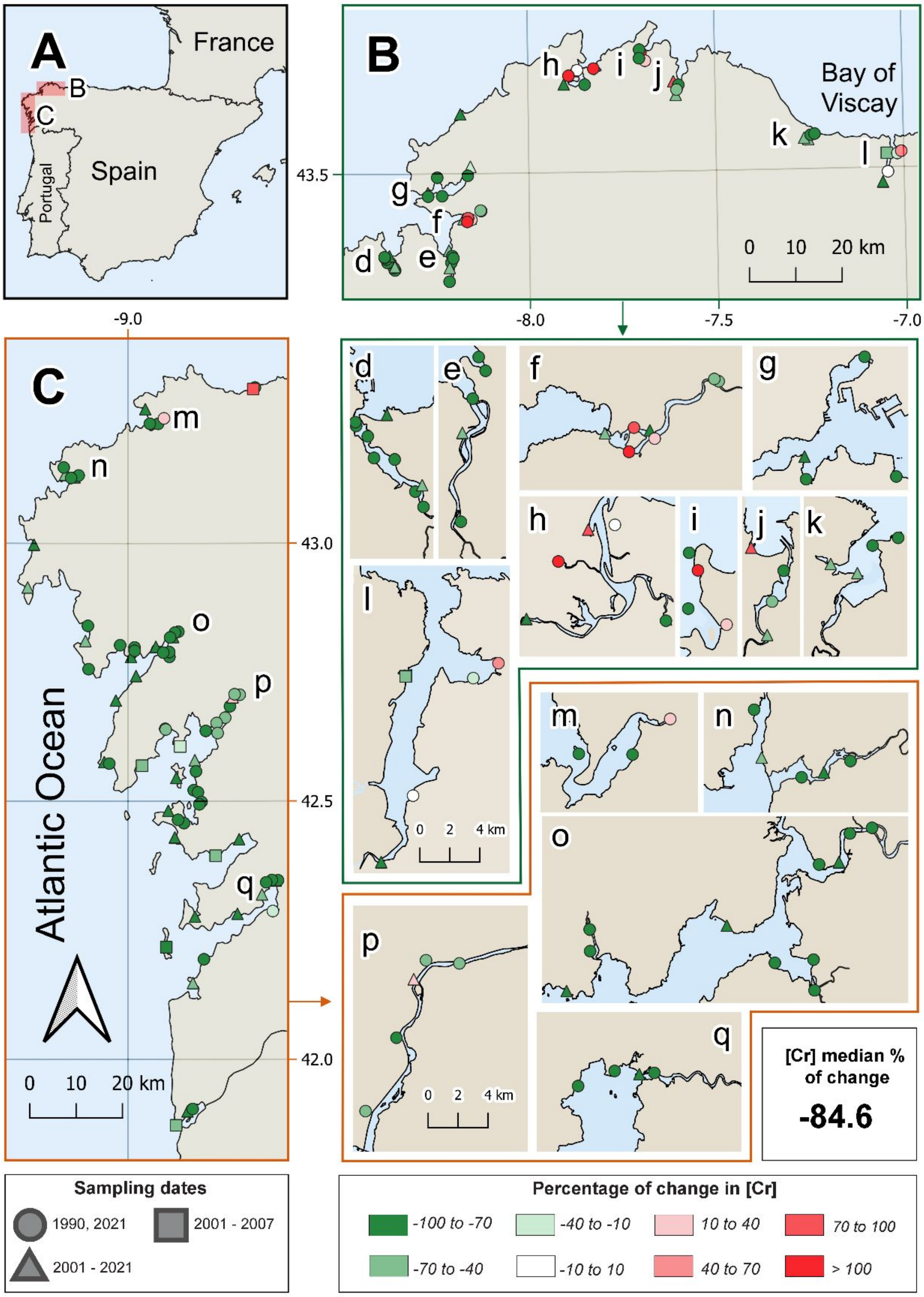

Fig. S15. Map of percentage changes in Cr concentrations over time. Panels A-C provide a regional overview, with B and C showing the differences between the final and initial Cr concentrations (in %) at each sampling site. Panels d-l and m-q offer detailed views

of stations that are densely clustered and hard to distinguish in B and C, respectively. Different colors represent the percentage changes in Cr concentrations, while distinct symbols indicate the sampling dates: 1990 and 2021, 2001-2021, and 2001-2007. The total median percentage change, calculated as the median of the percentage changes across all stations, is displayed below.

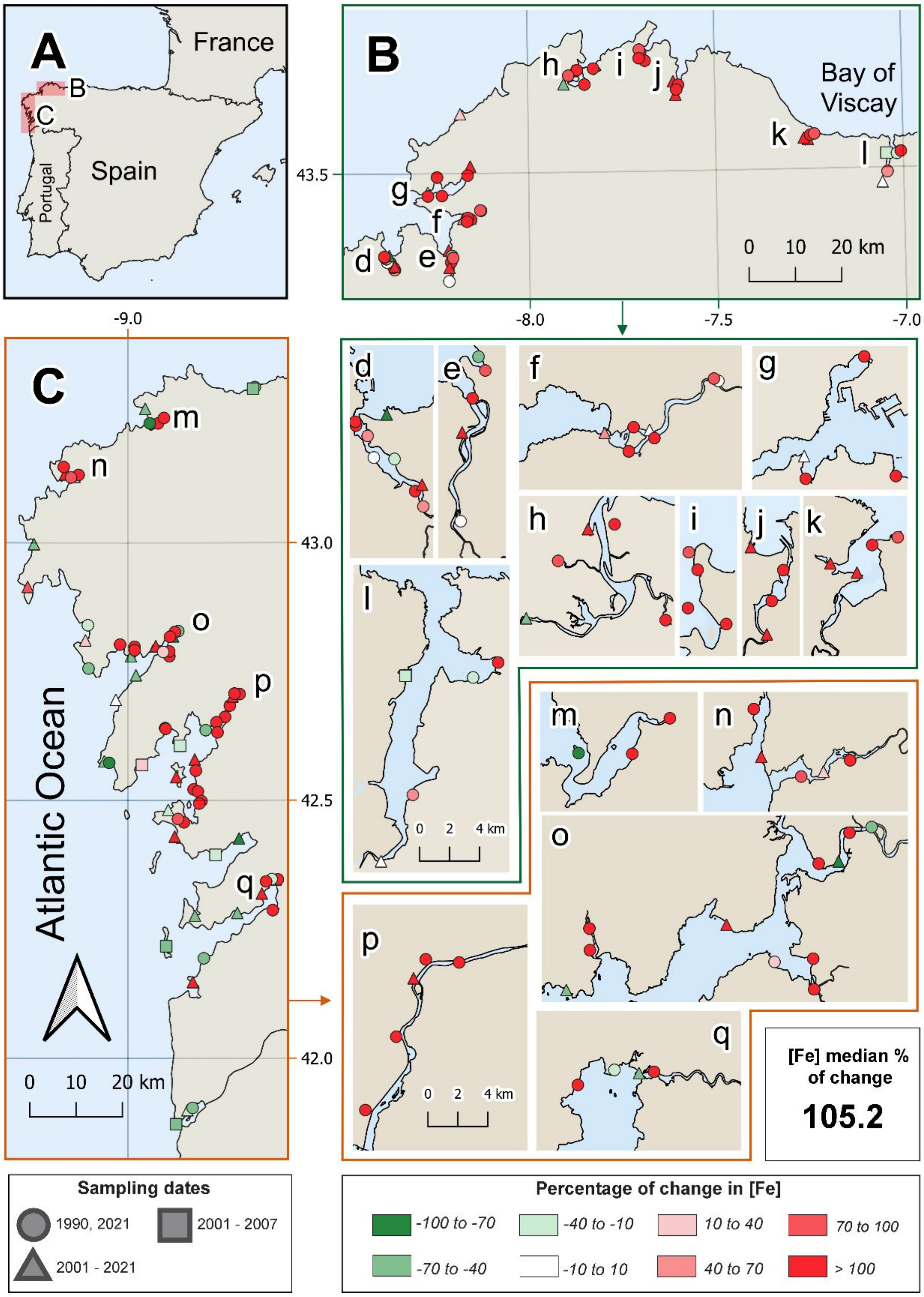

Fig. S16. Map of percentage changes in Fe concentrations over time. Panels A-C provide a regional overview, with B and C showing the differences between the final and initial Fe concentrations (in %) at each sampling site. Panels d-l and m-q offer detailed views

of stations that are densely clustered and hard to distinguish in B and C, respectively. Different colors represent the percentage changes in Fe concentrations, while distinct symbols indicate the sampling dates: 1990 and 2021, 2001-2021, and 2001-2007. The total median percentage change, calculated as the median of the percentage changes across all stations, is displayed below.

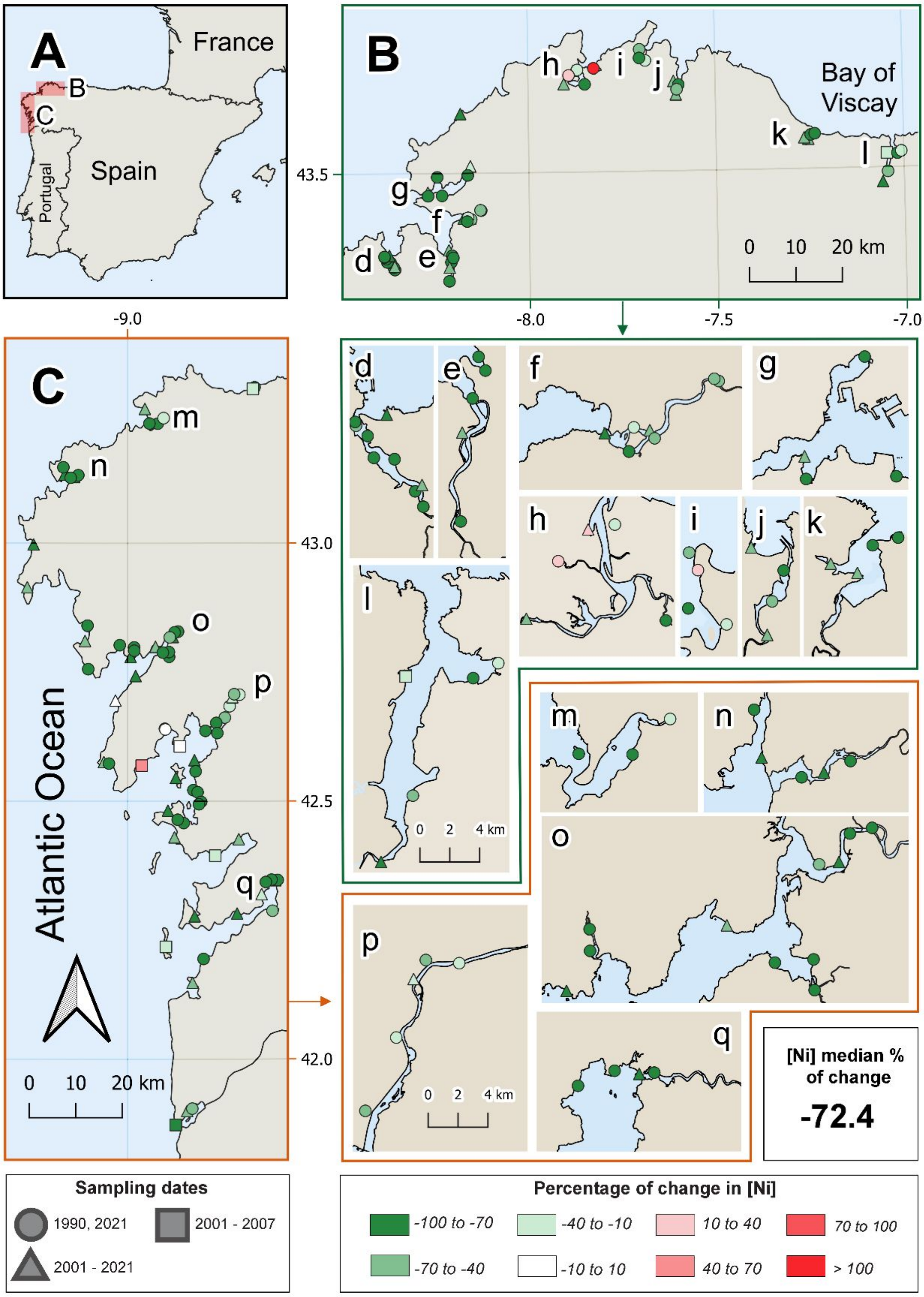

Fig. S17. Map of percentage changes in Ni concentrations over time. Panels A-C provide a regional overview, with B and C showing the differences between the final and initial Ni concentrations (in %) at each sampling site. Panels d-l and m-q offer detailed views

of stations that are densely clustered and hard to distinguish in B and C, respectively. Different colors represent the percentage changes in Ni concentrations, while distinct symbols indicate the sampling dates: 1990 and 2021, 2001-2021, and 2001-2007. The total median percentage change, calculated as the median of the percentage changes across all stations, is displayed below.

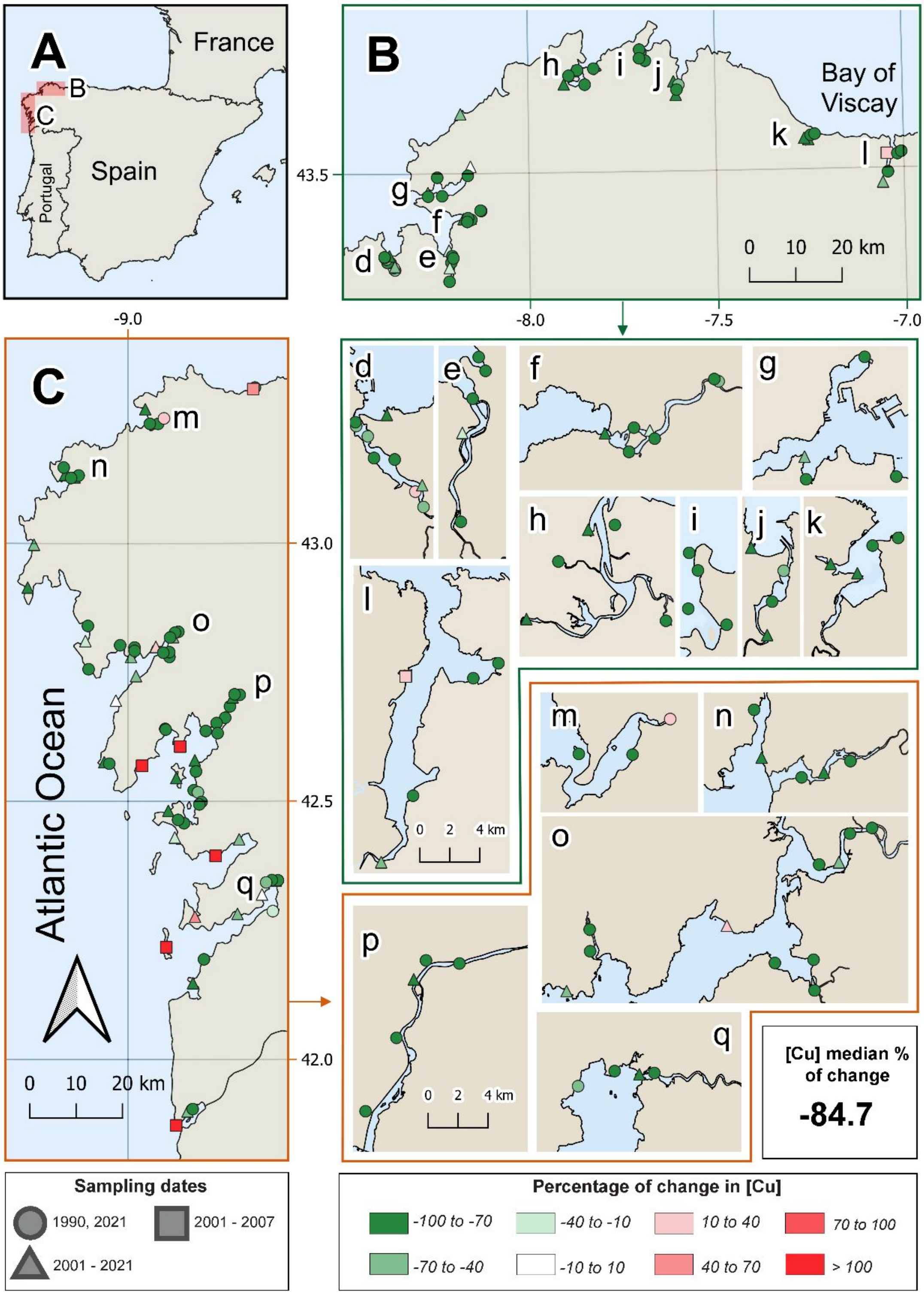

Fig. S18. Map of percentage changes in Cu concentrations over time. Panels A-C provide a regional overview, with B and C showing the differences between the final and initial Ni concentrations (in %) at each sampling site. Panels d-l and m-q offer detailed views

of stations that are densely clustered and hard to distinguish in B and C, respectively. Different colors represent the percentage changes in Ni concentrations, while distinct symbols indicate the sampling dates: 1990 and 2021, 2001-2021, and 2001-2007. The total median percentage change, calculated as the median of the percentage changes across all stations, is displayed below.

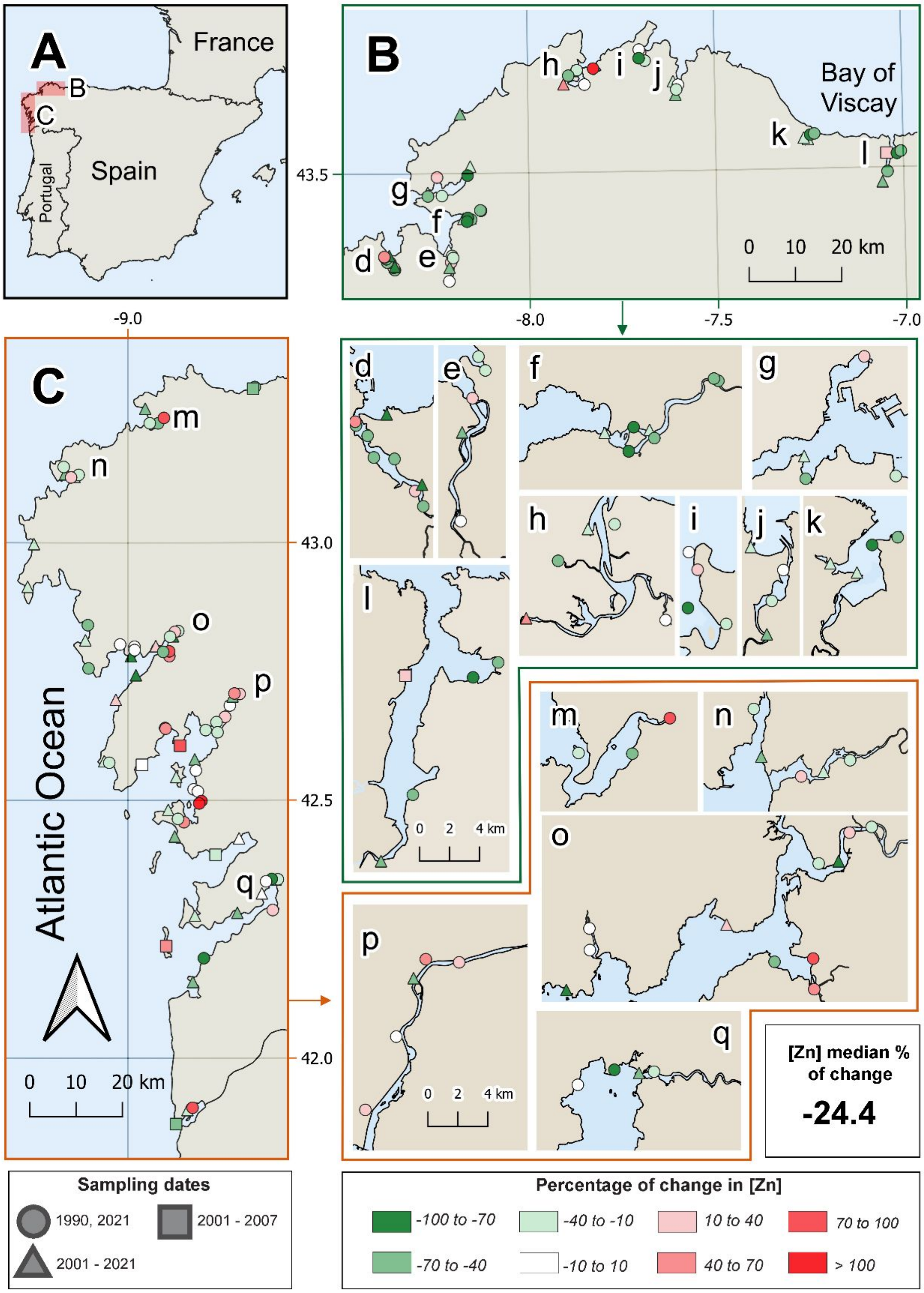

Fig. S19. Map of percentage changes in Zn concentrations over time. Panels A-C provide a regional overview, with B and C showing the differences between the final and initial Zn concentrations (in %) at each sampling site. Panels d-l and m-q offer detailed views

of stations that are densely clustered and hard to distinguish in B and C, respectively. Different colors represent the percentage changes in Zn concentrations, while distinct symbols indicate the sampling dates: 1990 and 2021, 2001-2021, and 2001-2007. The total median percentage change, calculated as the median of the percentage changes across all stations, is displayed below.

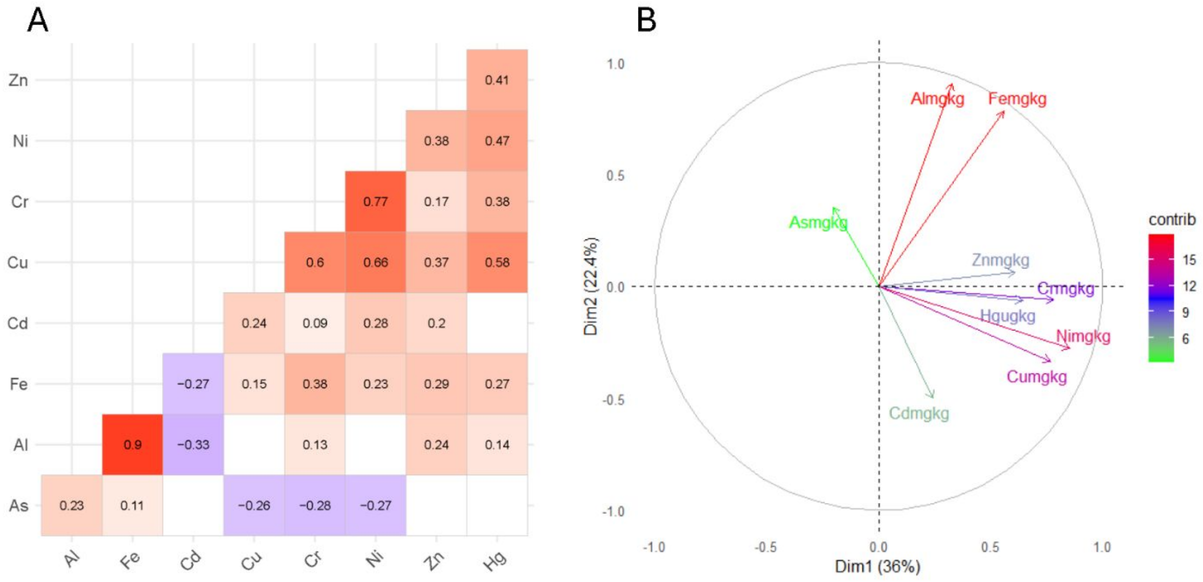

Fig. S20. Correlation matrix (A) and PCA (B) in PTEs concentrations in *Fucus* spp. A) Significant Spearman correlations (p<0.005, p-values adjusted using the Benjamini-Hochberg method) are indicated by displaying the correlation value. B) contribution loads of each PTE to the Dimension 1 and 2 are represented by color-coding.
